# The mediating roles of anthropo-metabolic biomarkers on the association between beverage consumption and breast cancer risk

**DOI:** 10.1186/s12937-025-01110-y

**Published:** 2025-03-22

**Authors:** Xiaoyi Lin, Boheng Liang, Tai Hing Lam, Kar Keung Cheng, Weisen Zhang, Lin Xu

**Affiliations:** 1 School of Public Health, Sun Yat-sen University, No. 74 Zhongshan 2nd Road, Guangzhou, Guangdong Province China; 2 Guangzhou Center for Disease Control and Prevention, Guangzhou, China; 3 School of Public Health, the University of Hong Kong, Hong Kong, China; 4 Guangzhou Twelfth People’s Hospital, Guangzhou, China; 5 School of Health Sciences, College of Medicine and Health, University of Birmingham, Birmingham, UK; 6 Greater Bay Area, Greater Bay Area Public Health Research Collaboration, Guangzhou, China

**Keywords:** Beverage consumption, Breast cancer prevention, Anthropo-metabolic biomarkers, Mediation, Sugar sweetened beverages

## Abstract

**Background:**

Breast cancer (BC) is the most common malignancy in women, yet the role of beverage consumption in BC risk remains unclear. Additionally, the contribution of anthropo-metabolic biomarkers as mediators is unknown, limiting the development of effective prevention strategies.

**Methods:**

This study included 13,567 participants from the Guangzhou Biobank Cohort Study (GBCS), where beverage consumption was assessed at baseline using a food frequency questionnaire. BC cases were identified through cancer registry linkage over a mean follow-up of 14.8 years. Mendelian randomization (MR) analyses were performed to evaluate the causal effects of beverage consumption on BC risk, with a two-step MR approach used to estimate mediation effects.

**Results:**

During follow-up, 243 BC cases were identified. Weekly consumption of ≥ 1 portion of sugar sweetened beverages (SSB), versus < 1 portion, was significantly associated with a higher risk of BC (hazard ratio [HR] 1.58, 95% confidence interval [CI] 1.12–2.23). This association was partly mediated by body mass index (proportion mediated [PM] 4.2%, 95% CI 0.9–17.1%) and uric acid (PM 18.8%, 95% CI 1.5–77.5%). Weekly consumption of > 6 portions of dairy-based milk was associated with a non-significantly higher BC risk (HR 1.41, 95% CI 0.99–2.03), while 3–6 portions of soy milk were associated with a lower BC risk (HR 0.31, 95% CI 0.10–0.98). No significant associations were found for pure fruit juice, coffee, tea, or alcoholic drinks. MR analyses supported the detrimental effect of SSB on BC risk, with high-density lipoprotein cholesterol, polyunsaturated fatty acids to total fatty acids (TFAs) ratio, and omega-6 fatty acids to TFAs ratio mediating 2.44%, 2.73%, and 3.53% of the association, respectively.

**Conclusion:**

This study suggested that SSB consumption was a risk factor for BC and identified key anthropo-metabolic biomarkers mediating this relationship. Reducing SSB consumption and addressing associated metabolic pathways may offer effective strategies for BC prevention.

**Supplementary Information:**

The online version contains supplementary material available at 10.1186/s12937-025-01110-y.

## Introduction

The global beverage consumption has increased over the past three decades [1–3], driven by its contribution to nutrient and energy intake, and hedonic appeal. This dietary trend has become a priority concern due to its association with adverse health outcomes, including mortality, cardiovascular disease, and cancer [4–6]. In China, the per capita beverage consumption is approximately 120 kg annually, which contributes to a high disease burden, with southern China being one of the regions most affected [7]. Patterns of beverage consumption vary across population subgroups, with women generally consuming fewer sugar sweetened beverages (SSB) and alcoholic drinks, but more pure fruit juice (PFJ) and milk than men [1, 8].

Breast cancer (BC) is the most common malignancy among women globally, accounting for nearly one-third of all newly diagnosed female cancers [9]. China accounts for about 18% of global BC cases [10, 11], driven by a rising incidence and an aging population [12]. Identifying modifiable risk factors, such as dietary habits including beverage consumption, is essential for developing effective prevention strategies. Previous studies showed that higher SSB consumption was associated with a higher risk of BC [13, 14], and alcohol consumption is an established BC risk factor [15]. However, evidence for other beverages, including milk [16, 17], PFJ [5, 13], tea [15, 18] and coffee [19] remains inconsistent, with some studies suggesting potential risks or protective effects, while others find no association.

The association between beverage consumption and BC risk may be mediated through metabolic dysregulation, including obesity, insulin resistance, and altered lipid metabolism, all of which can promote carcinogenesis [20–22]. Additionally, the anti-inflammatory and antioxidant properties of certain beverages may modulate oxidative stress, systemic inflammation, and hormonal pathways, which are key contributors to BC pathogenesis [23, 24]. However, no studies have specifically examined biological mediators that might explain these associations or could be targeted for intervention.

To address these gaps, we conducted a prospective cohort study and Mendelian randomization (MR) analysis, accompanied by mediation analyses to investigate the associations between beverage consumption and BC risk and to identify anthropo-metabolic biomarkers mediating these relationships. MR analysis uses genetic variants as instrumental variables, providing estimates that are less susceptible to confounding or reverse causation compared to conventional observation studies [25]. Mediation analysis additionally explores the intermediate variables through which an exposure influences an outcome [26].

## Methods

### Observational study

#### Study population

The Guangzhou Biobank Cohort Study (GBCS) is a three-way collaboration of the Guangzhou Twelfth People’s Hospital and the Universities of Hong Kong and Birmingham. Details have previously been described [27]. In brief, this longitudinal cohort consists of participants from the “Guangzhou Health and Happiness Association for the Respectable Elders” (GHHARE), a community social and welfare organization. The GHHARE included about 7% of Guangzhou permanent residents aged 50 years or older in all 10 districts of Guangzhou, the capital city of Guangdong province in southern China. GHHARE participants were eligible if they were ambulatory, capable of providing informed consent, and not undergoing treatments for life-threatening conditions. Only those recruited in phases 1 and 2 (2003–2006) were included in the current study, as the Food Frequency Questionnaire (FFQ) was shortened in phase 3 (2006–2008), limiting dietary exposure assessment.

Baseline information was collected using a computer-based standardized questionnaire by face-to-face interviews. The reproducibility of the questionnaire responses was tested by re-interviewing 200 randomly selected participants after a 1-month interval, which generated satisfactory results [27]. All laboratory analyses were performed on fresh blood samples in the Clinical Laboratory of the Guangzhou Twelfth People’s Hospital using standardized, automated, well-documented methodologies [27]. Physical examination was done by trained nurses in the hospital using standard protocols [27]. The Guangzhou Medical Ethics Committee of the Chinese Medical Association approved the study, and all participants provided written, informed consent before participation.

#### Exposures, outcome, and potential mediators

Baseline beverage consumption was assessed using a 300-item validated FFQ [28]. The FFQ included 19 commonly consumed beverages in Southern China, with intake calculated as the product of the number of portions per occasion and weekly frequency, expressed in portions per week (one portion = 250 mL). Beverages were categorized into seven types: dairy-based milk, soy milk, SSB, PFJ, coffee, tea, and alcoholic drinks (Table S1). Dairy-based milk and soy milk consumption were classified into four groups (< 1, 1–2, 3–6, > 6 portions/week), and SSB, PFJ, coffee, tea, and alcoholic drinks were classified into two groups (< 1, ≥ 1 portion/week) due to the relatively small number of high consumers.

Participants were followed until December 31, 2020, through linkage with the cancer registry and death registry of the Guangzhou Center for Disease Control and Prevention using their unique resident identity card numbers. BC cases were identified and coded as “C50” based on the International Statistical Classification of Diseases and Related Health Problems 10th Revision (ICD-10) by trained clinical coding officers at each hospital.

To investigate potential mechanisms, 12 anthropo-metabolic parameters previously associated with BC risk were assessed as mediators [29–33]. Anthropometric parameters included body mass index (BMI), waist circumference, and waist-to-hip ratio. BMI was calculated by dividing the weight (kg) by height squared (m^2^). Serum metabolic parameters included fasting glucose, lipids (total cholesterol, low-density lipoprotein cholesterol [LDL-C], high-density lipoprotein cholesterol [HDL-C], triglycerides), bilirubin, blood urea nitrogen, creatinine, and uric acid.

### Mendelian randomization

#### Instrument selection

Summary-level genome-wide association studies (GWAS) data for beverage intake (dairy-based milk, soy milk, SSB, PFJ, coffee, tea, and alcoholic drinks) were obtained from the UK Biobank, which used web-based 24-h recall questionnaire and touchscreen questionnaire at assessment center visit [34, 35]. Genetic instruments for BC were obtained from a meta-analysis of 67 GWAS studies including 122,977 cases and 105,974 controls [36]. Genetic instruments of 25 anthropo-metabolic markers, previously identified as BC risk predictors [29–33], were selected for mediation analyses [37–43]. Details about the GWAS data sources were summarized in Table S2.

Genetic instruments for each exposure and mediator were selected based on a genome-wide significant threshold (*P* < 5e-8), and independence criteria (linkage disequilibrium *r*^2^ < 0.001 within 10,000 kb). The thresholds for dairy-based milk, soy milk, SSB, PFJ, coffee, and tea were set at *P* < 5e-6 [34], due to the limited number of single nucleotide polymorphisms (SNPs) meeting the stringent criterion.

### Statistical analysis

#### Observational study

Baseline characteristics by BC status were compared using the t-test for continuous variables and Pearson's Chi-squared test for categorical variables. Potential confounders included age, socioeconomic position (education level, occupation, annual personal income), behavioral factors (smoking status, alcohol use, physical activity), reproductive factors (age at menarche and menopause, parity and breastfeeding history), personal and family medical history (oral contraceptive use, hormone replacement therapy, self-reported health status, and family history of BC), and daily dietary energy intake. Beverage consumption was analyzed both as a categorical and a continuous variable. Cox proportional hazards regression was used to estimate the association between beverage consumption and BC risk, yielding crude and adjusted hazard ratios (HRs) and 95% confidence intervals (CIs). The proportional hazards assumption was assessed using the Schoenfeld residual test, with no violation detected. Stratified analyses were conducted by age (< 60/ ≥ 60 years) and menopausal status. Sensitivity analyses were performed. First, to minimize reverse causality, analyses were repeated after excluding BC cases or deaths occurring within the first year of follow-up. Second, to account for competing risks, proportional subdistribution hazards regression was used to estimate the subdistribution HRs [44]. Third, four partially adjusted models were constructed, each accounting for a specific set of potential confounders including socioeconomic position, behavioral factors, reproductive factors, and personal and family medical history, to assess the influence of these various confounder categories.

Linear regression was used to estimate the associations between beverage consumption and anthropo-metabolic parameters, with the assumptions of linearity, normality of residuals, homoscedasticity, and independence being tested and verified. Significant markers were subsequently included in mediation analyses. Mediation proportions were estimated using the difference method by comparing regression models with and without the inclusion of the mediator [45, 46].

#### Mendelian randomization

Instrument strength was evaluated using the F-statistic, with a value > 10 considered sufficient to minimize weak instrument bias. Harmonization of genetic effects was performed prior to analysis to ensure consistency in directionality between exposure and outcome associations. For each independent SNP (*r*^2^ < 0.001), the Wald ratio was calculated as the SNP-outcome association divided by the SNP-exposure association. These estimations were combined using inverse-variance weighted (IVW) with random effects independent [47]. Heterogeneity among SNPs was assessed using Cochran’s Q test [48]. The weighted median, MR-Egger, and MR pleiotropy residual sum and outlier (MR-PRESSO) methods were performed to validate the robustness of the IVW results based on different assumptions.

Univariable MR estimated the total effect of the exposure on the outcome. Then, two-step MR was used to estimate the indirect effect of the exposure on the outcome through a mediator [26]. Univariable MR was first used to estimate the causal effect of the exposure on the mediator and the causal effect of the mediator on the outcome. The indirect effect was calculated by multiplying these two estimates, following the product-of-coefficients method. The delta method was used to approximate the CIs of mediation effects [49]. Negative mediation proportions were truncated at 0%, as this is the minimum threshold for determining mediation. IVW was used as the primary method for estimating mediating effects.

Statistical significance was defined as a two-sided *P*-value < 0.05. Observational mediation analyses were conducted using the %MEDIATE Macro in SAS (version 9.4; SAS Institute, Cary, NC). All other statistical analyses were conducted using R software (version 4.3.1).

## Results

### Baseline characteristics

Among GBCS participants (phase 1–2) who completed the FFQ, participants with a self-reported or documented cancer diagnosis, those lost to follow-up, male participants, and those with missing data on confounders were excluded, yielding an analytical sample of 13,567 participants (Figure S1). During an average follow-up period of 14.8 years with 201,277 person-years, 243 BC cases were identified. Table 1 shows that participants who developed BC were younger, had higher education levels and annual personal income, and a greater proportion of non-manual workers (all *P*-values < 0.05). Additionally, the BC group also had a lower prevalence of active physical activity and a higher proportion of consuming ≥ 1 portion of SSB per week (all *P*-values < 0.05).

**Table 1:** Baseline characteristics of participants in the Guangzhou Biobank Cohort Study

	**Breast cancer**		
**Characteristics**	**No (*N* = 13,324)**	**Yes (*N* = 243)**	***P*-value**
Age, years (mean ± SD)	61.92 ± 6.63	61.05 ± 6.63	0.04
**Socioeconomic position**			
Education level			< 0.001
Primary or below	6,900 (52%)	92 (38%)	
Middle school or above	6,424 (48%)	151 (62%)	
Occupation			< 0.001
Manual	8,817 (66%)	133 (55%)	
Non-manual	2,574 (19%)	56 (23%)	
Others	1,933 (15%)	54 (22%)	
Personal income, RMB/year			0.01
< 10,000	5,726 (43%)	87 (36%)	
≥ 10,000	6,935 (52%)	149 (61%)	
Not reported	663 (5.0%)	7 (2.9%)	
**Behavioral factors**			
Smoking status			0.40
Never	12,798 (96%)	236 (97%)	
Former or current	526 (3.9%)	7 (2.9%)	
Alcohol use			0.99
Never	11,949 (90%)	218 (90%)	
Former or current	1,375 (10%)	25 (10%)	
Physical activity			0.01
Inactive/Moderate	7,274 (55%)	152 (63%)	
Active	6,050 (45%)	91 (37%)	
**Reproductive factors**			
Age at menarche, years			0.08
> 12	12,303 (92%)	217 (89%)	
≤ 12	1,021 (7.7%)	26 (11%)	
Age at menopause, years			0.30
< 45	1,187 (8.9%)	17 (7.0%)	
≥ 45	12,137 (91%)	226 (93%)	
Parity, no	1,312 (9.8%)	15 (6.2%)	0.06
Breastfeeding history, no	1,733 (13%)	26 (11%)	0.29
**Personal and family medical history**			
Oral contraceptive use, yes	2,299 (17%)	46 (19%)	0.49
Hormone replacement therapy, yes	145 (1.1%)	4 (1.6%)	0.61
Self-reported health status, poor	2,566 (19%)	59 (24%)	0.05
Family history of breast cancer, yes	105 (0.8%)	4 (1.6%)	0.26
**Dietary factors**			
Daily dietary energy intake, kcal (mean ± SD)	1,757.74 ± 497.76	1,769.28 ± 526.81	0.74
Dairy-based milk			0.05
< 1 portion/week	9,222 (69%)	151 (62%)	
1–2 portions/week	1,163 (8.7%)	25 (10%)	
3–6 portions/week	1,469 (11%)	28 (12%)	
> 6 portions/week	1,470 (11%)	39 (16%)	
Soy milk			0.18
< 1 portion/week	11,308 (85%)	210 (86%)	
1–2 portions/week	1,355 (10%)	28 (12%)	
3–6 portions/week	493 (3.7%)	3 (1.2%)	
> 6 portions/week	168 (1.3%)	2 (0.8%)	
Sugar sweetened beverages			0.003
< 1 portion/week	11,869 (89%)	202 (83%)	
≥ 1 portion/week	1,455 (11%)	41 (17%)	
Pure fruit juice			0.44
< 1 portion/week	12,989 (97%)	235 (97%)	
≥ 1 portion/week	335 (2.5%)	8 (3.3%)	
Coffee			0.39
< 1 portion/week	13,046 (98%)	236 (97%)	
≥ 1 portion/week	278 (2.1%)	7 (2.9%)	
Tea			0.92
< 1 portion/week	9,611 (72%)	176 (72%)	
≥ 1 portion/week	3,713 (28%)	67 (28%)	
Alcoholic drinks			0.40
< 1 portion/week	13,045 (98%)	236 (97%)	
≥ 1 portion/week	279 (2.1%)	7 (2.9%)	

*Abbreviations: SD*, standard deviation. 1 portion = 250 ml

### Association between beverage consumption and BC risk

Table 2 shows that, after adjusting for 16 potential confounders, the consumption of > 6 portions of dairy-based milk per week was marginally associated with an increased risk of BC (HR 1.41, 95% CI 0.99–2.03, *P* = 0.06), compared with < 1 portion per week. In contrast, participants consuming 3–6 portions of soy milk per week showed a marginally lower risk of BC (HR 0.31, 95% CI 0.10–0.98, *P* = 0.047). For SSB, consumption of ≥ 1 portion per week was significantly associated with an increased risk of BC (HR 1.58, 95% CI 1.12–2.23, *P* = 0.009), with each additional portion associated with a 5% higher risk of BC (95% CI 1.03–1.07, *P* < 0.001). No significant associations were observed between BC risk and the consumption of PFJ, coffee, tea, or alcoholic drinks.

**Table 2:** Associations between beverage consumption and risk of breast cancer in the Guangzhou Biobank Cohort Study

	**N**	**Incidence rate/1000 person-years**	**Crude HR (95% CI)**	**Adjusted HR (95% CI)**^**a**^	***P***^**b**^
**Dairy-based milk**					
< 1 portion/week	9,373	1.08	1.00	1.00	
1–2 portions/week	1,188	1.44	1.33 (0.87, 2.03)	1.15 (0.75, 1.77)	0.51
3–6 portions/week	1,497	1.26	1.16 (0.77, 1.74)	0.98 (0.65, 1.48)	0.94
> 6 portions/week	1,509	1.76	1.62 (1.14, 2.31)	1.41 (0.99, 2.03)	0.06
per 1 portion			1.05 (1.01, 1.09)	1.03 (0.99, 1.08)	0.15
**Soy milk**					
< 1 portion/week	11,518	1.23	1.00	1.00	
1–2 portions/week	1,383	1.35	1.09 (0.74, 1.62)	1.04 (0.70, 1.54)	0.87
3–6 portions/week	496	0.39	0.32 (0.10, 0.99)	0.31 (0.10, 0.98)	0.047
> 6 portions/week	170	0.79	0.64 (0.16, 2.59)	0.63 (0.16, 2.54)	0.52
per 1 portion			0.89 (0.77, 1.03)	0.88 (0.76, 1.02)	0.09
**Sugar sweetened beverages**					
< 1 portion/week	12,071	1.13	1.00	1.00	
≥ 1 portion/week	1,496	1.88	1.67 (1.19, 2.33)	1.58 (1.12, 2.23)	0.009
per 1 portion			1.05 (1.03, 1.07)	1.05 (1.03, 1.07)	< 0.001
**Pure fruit juice**					
< 1 portion/week	13,224	1.20	1.00	1.00	
≥ 1 portion/week	343	1.57	1.31 (0.65, 2.65)	1.22 (0.60, 2.49)	0.57
per 1 portion			1.13 (0.95, 1.34)	1.13 (0.94, 1.36)	0.18
**Coffee**					
< 1 portion/week	13,282	1.20	1.00	1.00	
≥ 1 portion/week	285	1.69	1.42 (0.67, 3.01)	1.19 (0.56, 2.54)	0.65
per 1 portion			0.87 (0.56, 1.35)	0.82 (0.52, 1.29)	0.39
**Tea**					
< 1 portion/week	9,787	1.21	1.00	1.00	
≥ 1 portion/week	3,780	1.19	0.98 (0.74, 1.30)	1.02 (0.77, 1.35)	0.90
per 1 portion			1.00 (0.99, 1.01)	1.00 (0.99, 1.01)	0.65
**Alcoholic drinks**					
< 1 portion/week	13,281	1.20	1.00	1.00	
≥ 1 portion/week	286	1.69	1.42 (0.67, 3.01)	1.22 (0.56, 2.62)	0.62
per 1 portion			1.00 (0.82, 1.22)	0.99 (0.73, 1.34)	0.93

*Abbreviations*: *HR* hazard ratio, *CI* confidence interval. 1 portion = 250 ml

^a^Adjusted for age, education level, occupation, annual personal income, smoking status, alcohol use, physical activity, age at menarche and menopause, parity and breastfeeding history, oral contraceptive use, hormone replacement therapy, self-reported health status, family history of breast cancer, and daily dietary energy intake

^b^*P*-value was for the adjusted model

A significant interaction between SSB consumption and age was observed, with the positive association between SSB consumption and BC risk being statistically significant only in participants younger than 60 years (Table S3). Stratification by menopausal status showed that this association was significant only in postmenopausal women (Table S4). In sensitivity analyses, the positive association between SSB consumption and BC risk remained after excluding BC cases or deaths occurring within the first year of follow-up (Table S5). Results from the competing risk analysis were consistent with the main analysis (Table S6). The association of SSB consumption with BC risk remained across all partially adjusted models (Table S7).

### Mediation analysis

**Fig. 1:**
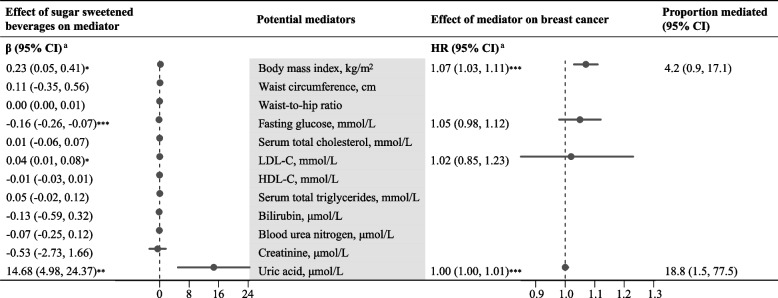
Mediating effects of the association between sugar sweetened beverages and risk of breast cancer by anthropo-metabolic markers in the Guangzhou Biobank Cohort Study. Abbreviations: CI, confidence interval; HR, hazard ratio; LDL-C, low-density lipoprotein cholesterol; HDL-C, high-density lipoprotein cholesterol. ^a^Adjusted for age, education level, occupation, annual personal income, smoking status, alcohol use, physical activity, age at menarche and menopause, parity and breastfeeding history, oral contraceptive use, hormone replacement therapy, self-reported health status, family history of breast cancer, and daily dietary energy intake. *P < 0.05; **P < 0.01; ***P < 0.001

For the mediation analysis, we further restricted to 13,359 participants with complete data on BMI, waist circumference, waist-to-hip ratio, fasting glucose, serum total cholesterol, LDL-C, and HDL-C, serum total triglycerides, and to 6,855 participants with additional data on total bilirubin, blood urea nitrogen, serum creatinine, and uric acid (Figure S1). Table S8 shows that compared with < 1 portion/week, consumption of 3–6 portions of soy milk per week was significantly associated with higher levels of BMI and waist circumference (all *P*-values < 0.01). Consumption of ≥ 1 portion of SSB was significantly associated with higher levels of BMI, LDL-C, and uric acid, but lower levels of fasting glucose (all *P*-values < 0.05). In Fig. 1, mediation analysis identified BMI and uric acid as mediators of the association of SSB consumption with higher BC risk, with a mediation proportion of 4.2% (95% CI 0.9–17.1%, *P* = 0.007) and 18.8% (95% CI 1.5–77.5%, *P* = 0.02), respectively, and a total mediation effect of 22.4% (95% CI 1.7–83.0%, *P* = 0.006). No significant mediation was observed for the association between soy milk consumption and BC risk (Table S9).

### Mendelian randomization

#### Causal effect of beverage consumption on BC

A total of 20, 27, 14, 9, 81, 86, and 77 SNPs were selected as instruments for dairy-based milk, soy milk, SSB, PFJ, coffee, tea, and alcoholic drinks consumption, respectively. The average F-statistics for these instruments were 24.3, 23.2, 24.1, 21.8, 44.7, 31.8 and 62.3. Table S10 summarizes the genetic instruments used for beverage consumption.

Table 3 shows that higher genetically determined SSB consumption was associated with an increased BC risk (odds ratio [OR] 3.52, 95% CI 1.06–11.70, *P* = 0.04). The MR-PRESSO method identified two SNPs as influential outliers, and the outlier-corrected estimates remained statistically significant (OR 1.98, 95% CI 1.48–2.66, *P* < 0.001). The direction of the association was consistent across all four MR methods, although weighted median and MR-Egger results had wider CIs. No evidence for directional horizontal pleiotropy was detected (*P* for MR-Egger intercept = 0.75). However, genetically determined consumption of dairy-based milk, soy milk, PFJ, coffee, tea, and alcoholic drinks were not associated with BC risk in any MR analysis. Regarding BC subtypes (Table S11), genetically determined SSB consumption was consistently associated with an increased risk of estrogen receptor (ER)-negative BC (OR 5.69, 95% CI 1.22–26.63, *P* = 0.03), and was marginally associated with an increased risk of ER-positive BC (OR 3.07, 95% CI 0.94–9.98, *P* = 0.06).

**Table 3:** Two-sample Mendelian randomization estimates for the causal associations between beverage consumption and overall breast cancer

Exposure	SNP	F statistic	Methods	OR (95% CI)	*P*	Cochran’s Q statistic (I^2^)	MR-Egger Intercept (*P*)
Dairy-based milk	20	24.3	IVW	1.26 (0.75, 2.12)	0.38	14.54 (0.0%)	-0.002 (0.77)
			WM	1.06 (0.51, 2.20)	0.87		
			MR-Egger	1.63 (0.28, 9.47)	0.60		
			MR-PRESSO	-	-		
Soy milk	27	23.2	IVW	0.98 (0.92, 1.05)	0.59	35.76 (27.3%)	-0.002 (0.81)
			WM	0.96 (0.88, 1.05)	0.39		
			MR-Egger	1.01 (0.82, 1.24)	0.96		
			MR-PRESSO	-	-		
Sugar sweetened beverages	14	24.1	IVW	3.52 (1.06, 11.70)	0.04	85.10 (84.7%)	0.006 (0.75)
			WM	1.60 (0.81, 3.17)	0.18		
			MR-Egger	1.99 (0.05, 80.07)	0.72		
			MR-PRESSO^a^	1.98 (1.48, 2.66)	< 0.001		
Pure fruit juice	9	21.8	IVW	3.07 (0.38, 24.94)	0.29	83.49 (90.4%)	0.030 (0.31)
			WM	0.99 (0.41, 2.40)	0.98		
			MR-Egger	0.24 (0.00, 36.10)	0.59		
			MR-PRESSO^a^	0.85 (0.51, 1.42)	0.55		
Coffee	81	44.7	IVW	0.87 (0.67, 1.13)	0.29	301.33 (73.5%)	-0.004 (0.28)
			WM	0.92 (0.71, 1.20)	0.54		
			MR-Egger	1.12 (0.66, 1.88)	0.68		
			MR-PRESSO^a^	0.84 (0.68, 1.04)	0.11		
Tea	86	31.8	IVW	1.07 (0.88, 1.31)	0.50	172.71 (50.8%)	-0.003 (0.35)
			WM	1.02 (0.81, 1.28)	0.87		
			MR-Egger	1.43 (0.76, 2.70)	0.27		
			MR-PRESSO^a^	0.92 (0.80, 1.06)	0.23		
Alcoholic drinks	77	62.3	IVW	1.02 (0.88, 1.19)	0.79	262.73 (71.1%)	0.001 (0.69)
			WM	0.95 (0.83, 1.09)	0.50		
			MR-Egger	0.98 (0.76, 1.26)	0.87		
			MR-PRESSO^a^	0.99 (0.89, 1.11)	0.91		

*Abbreviations*: SNP, single nucleotide polymorphism; IVW, inverse-variance weighted; WM, weighted median method; MR-PRESSO, Mendelian randomization pleiotropy residual sum and outlier; OR, odds ratio; CI, confidence interval

^a^2SNPs were identified as influential outliers when consumption of sugar sweetened beverages was exposure: rs2472297, rs55872725; 1 SNP was identified as influential outliers when consumption of pure fruit juice was exposure: rs9972653; 7 SNPs were identified as influential outliers when consumption of coffee was exposure: rs10865548, rs111994577, rs2231142, rs2472297, rs2521501, rs57918684, rs9937053; 4 SNPs were identified as influential outliers when consumption of tea was exposure: rs11715828, rs1481012, rs2074551, rs2271961; 4 SNPs were identified as influential outliers when consumption of alcoholic drinks was exposure: rs12030672, rs2959005, rs56094641, rs62244890

#### Mediation analysis

**Fig. 2:**
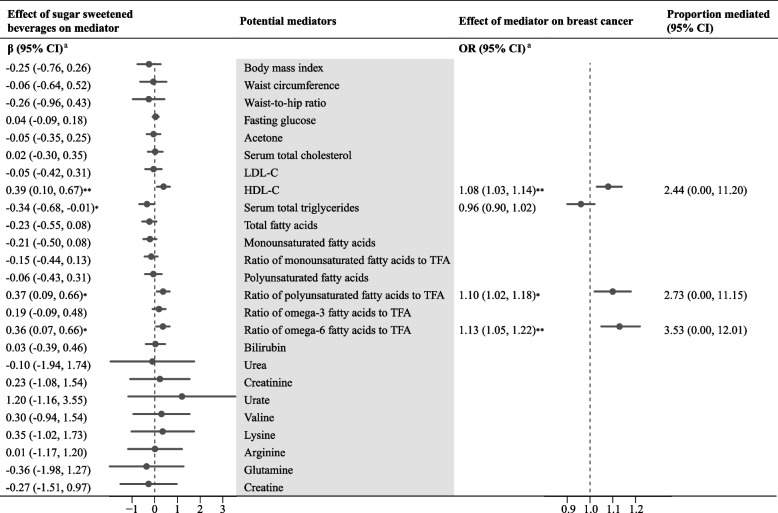
Mediation analysis on the causal association between sugar sweetened beverages and breast cancer using two-step Mendelian randomization. Abbreviations: CI, confidence interval; OR, odds ratio; LDL, low-density lipoprotein; HDL-C, high-density lipoprotein cholesterol; TFAs, total fatty acids. .^a^The β/OR and 95% CI were estimated using two-sample univariable Mendelian randomization analysis, with inverse-variance weighted as the main method. *P < 0.05; **P < 0.01

Among 25 anthropo-metabolic biomarkers (Table S12), genetically determined higher consumption of SSB was causally associated with HDL-C, the ratio of polyunsaturated fatty acids (PUFAs) to total fatty acids (TFAs), the ratio of omega-6 fatty acids to TFAs, and lower total triglycerides (all *P*-values < 0.05). No evidence of directional horizontal pleiotropy was observed (all *P*-values for MR-Egger intercept ≥ 0.33). For these four potential mediators (Table S13), genetically determined HDL-C, ratio of PUFAs to TFAs, and ratio of omega-6 fatty acids to TFAs were positively associated with an increased risk of BC (all *P*-values < 0.05). Instrumental validity test confirmed sufficient instrument strength for these biomarkers (all F-statistics > 110), with no evidence for horizontal pleiotropy (all *P* for MR-Egger intercept ≥ 0.06). Following mediator selection, two-step MR analysis showed that HDL-C, the ratio of PUFAs to TFAs, and the ratio of omega-6 fatty acids to TFAs mediated 2.44%, 2.73%, and 3.53% of the total effect of SSB consumption on BC risk, respectively (Fig. 2).

## Discussion

This study, triangulating evidence from observational and MR analyses, showed that higher SSB consumption was causally associated with an increased BC risk. Mediation analyses identified key anthropo-metabolic biomarkers, including BMI, uric acid, HDL-C, ratio of PUFAs to TFAs, and ratio of omega-6 fatty acids to TFAs as mediators of this association. Notably, these mediators explained a substantial proportion of the total effect of SSBs on BC risk, highlighting potential biological pathways that linked dietary habits to cancer risk. Furthermore, while dairy-based milk and soy milk showed marginal associations with BC risk, no significant associations were observed for PFJ, coffee, tea, or alcohol consumption. These findings indicated the potential causal links between SSB consumption and BC risk, and suggested the importance of addressing specific metabolic mediators, such as BMI and uric acid, in developing targeted cancer prevention strategies.

### Comparison with other studies

Our findings were consistent with the NutriNet-Santé prospective cohort, which identified a positive association between sugary drink consumption and BC risk in adults aged ≥ 18 years [5]. However, this study used an extensive dietary assessment approach, incorporating nearly 100 sugary drink items and repeated dietary measurements over time, which enhances dietary exposure assessment precision but may limit generalizability to other populations. Similarly, another prospective study conducted on middle-aged university graduates reported a positive association between sugary drink consumption and BC risk, but only in postmenopausal women, not in premenopausal women [50]. In contrast, the Nurses’ Health Study and the Canadian Study of Diet, Lifestyle, and Health reported no association between SSB consumption and BC risk when using < 1/month or none as the reference group [51, 52]. These discrepancies may reflect differences in study design, including the definitions of SSB exposure and variation in population characteristics. For example, the null findings in the Nurses’ Health Study and the Canadian cohort may be attributed to the younger participant profiles with mean ages < 50 years, which could lower the overall BC risk and attenuate the association with SSB consumption [51, 52]. Regarding milk consumption, no association with BC risk was reported in a cohort of over 60,000 female participants [53]. However, dairy milk intake at the 90th percentile was associated with an increased BC risk in a cohort of more than 50,000 women [54]. Furthermore, a previous MR study using a single SNP near the lactase gene locus as a proxy for milk consumption reported a positive association between milk consumption and BC risk [17]. These discrepancies may be due to variations in milk consumption classifications and genetic predispositions related to dairy digestion. Our findings on soy milk consumption were consistent with a study that analyzed soy milk intake as a continuous variable, which also found no significant linear association with BC risk [54]. However, that study reported that substituting dairy milk intake with soy milk was associated with a lower BC risk, suggesting a potential protective effect when replacing specific dietary components rather than from soy milk alone [54]. Our results for PFJ and tea consumption were also consistent with findings from large-scale cohort studies, which reported no significant associations with BC risk [5, 19]. For coffee, our findings were consistent with an American study that found no association between coffee intake and postmenopausal BC risk [55]. However, a European cohort study that differentiated between caffeinated and decaffeinated coffee suggested that higher caffeinated coffee intake may be associated with a lower risk of postmenopausal BC [19]. These discrepancies highlight the potential influence of coffee type and preparation methods on cancer risk, warranting further investigation. Regarding alcoholic drinks, our findings aligned with those of a Japanese study reporting no association between alcohol consumption and BC risk among Asian postmenopausal women [56]. Given the relatively low alcohol intake among Chinese women and the smaller sample size, our study may not have sufficient statistical power to detect a potential association. Furthermore, the low prevalence of obesity in our population could attenuate the effects of alcohol-induced estrogen production from adipose tissue [56, 57].

### Mechanisms

The association between SSB consumption and BC risk is most commonly attributed to obesity [20, 58], supported by our study which identified BMI as a potential mediator. Chronic SSB intake contributes to weight gain through excessive caloric intake and metabolic dysregulation, establishing a link between BMI and BC risk. Furthermore, uric acid emerged as an additional mediator. Fructose metabolism in SSB promotes ATP degradation and de novo purine synthesis, leading to elevated uric acid levels [59–61]. While evidence regarding the direct association between uric acid and BC risk remains inconclusive [62, 63], uric acid is a known inducer of oxidative stress, potentially driving the malignant transformation of breast cells [64, 65]. Additionally, uric acid may act as a secondary mediator, linking BMI to BC risk [33], as higher BMI can impair renal tubular excretion of uric acid in the context of SSB consumption [66]. In addition to BMI and uric acid, our MR analysis indicated a positive causal association of HDL-C with BC risk, consistent with previous study [67], However, this finding contrasted with observational evidence showing SSB consumption was associated with lower HDL-C levels [68]. This discrepancy underscores the complexity of lipid metabolism in relation to SSB intake and BC risk, highlighting the need for further investigation to clarify these relationships. Alterations in fatty acid metabolism may represent another pathway linking SSB to BC. Evidence suggests that increased basal hepatic fatty acids synthesis is one of the earliest metabolic changes induced by SSB consumption, preceding hypertriglyceridemia, hyperglycemia, or hyperinsulinemia [69]. Additionally, SSB consumption may alter PUFA metabolism through changes in acylcarnitine production and alterations in β-oxidation flux [70, 71]. Although limited evidence implicates PUFAs, particularly omega-6 fatty acids, in BC development [72, 73], the role of omega-6 fatty acids as potential mediators of SSB consumption and BC risk warrants further investigations.

### Strengths and limitations of this study

The major strength of our study was the use of two study designs, which yielded consistent findings. Moreover, this study provides the first evidence identifying anthropometric and metabolic mediators, such as BMI and uric acid, linking SSB consumption to BC risk. These findings highlight potential modifiable biomarkers that could inform prevention strategies for SSB-related BC. However, there are several limitations. First, beverage consumption was measured at a single time point, which might not fully capture long-term exposure. Nevertheless, the MR reflects lifelong differences in usual levels of exposure mitigating this concern [74]. Second, while extensive sensitivity analyses were performed, the possibility of pleiotropic effects cannot be entirely ruled out. For example, genetic variants associated with SSB consumption might also influence other dietary habits, introducing potential pleiotropy. Although observational analyses adjusted for multiple potential confounders, residual or unmeasured confounding could not be excluded. Therefore, further evidence is required to definitively establish causality. Third, our cohort study consisted exclusively of older Chinese women, which limits the generalizability of the findings to younger or more diverse populations. However, older women tend to have slower metabolism and reduced appetite [75], and SSB consumption is less prevalent in this demographic, potentially leading to an underestimation of the true association. Finally, the mediation analyses focused on widely recognized biomarkers, which may not fully capture all relevant pathways. Future studies should investigate additional potential mediators, to provide a more comprehensive understanding of the mechanisms underlying the observed associations.

## Conclusion

Both observational and MR studies identified SSB consumption as a significant risk factor for BC, mediated by modifiable anthropo-metabolic markers such as BMI and uric acid. Our findings suggested a potential basis for targeted prevention strategies, implying that reducing SSB intake and addressing associated metabolic alternations may help mitigate BC risk.

## Supplementary Information

Additional file 1: Table S1. Beverage items used in the analysis. Table S2. Overview of genome-wide association study data. Table S3. Associations between beverage consumption and risk of breast cancer stratified by baseline age in the Guangzhou Biobank Cohort Study. Table S4. Associations between beverage consumption and risk of breast cancer stratified by baseline menopausal status in the Guangzhou Biobank Cohort Study. Table S5. Associations between beverage consumption and risk of breast cancer in 13,534 participants, excluding breast cancer or death events within the first year of follow-up in the Guangzhou Biobank Cohort Study. Table S6. Associations between beverage consumption and risk of breast cancer in the Guangzhou Biobank Cohort Study by proportional subdistribution hazards regression. Table S7. Associations between beverage consumption and risk of breast cancer in the Guangzhou Biobank Cohort Study using four partially adjusted models. Table S8. Associations between beverage consumption and 16 baseline anthropo-metabolic markers in the Guangzhou Biobank Cohort Study. Table S9. Mediation analysis with anthropo-metabolic markers as potential mediators for the association between beverage consumption and risk of breast cancer in the Guangzhou Biobank Cohort Study. Table S10. Summary information of the single nucleotide polymorphisms (SNPs) used as instrumental variables for beverage consumption in univariable Mendelian randomization. Table S11. Mendelian randomization estimates for the causal associations between sugar sweetened beverages consumption and estrogen receptor (ER)-positive/negative breast cancer. Table S12. Mendelian randomization estimates for the causal associations between sugar sweetened beverages consumption and anthropo-metabolic biomarkers. Table S13. Mendelian randomization estimates for the causal associations between anthropo-metabolic biomarkers and overall breast cancer. Figure S1. Flow diagram of participants selection.

## Data Availability

(1) Observational study: Data that support findings are not publicly available due to the protection of the privacy of participants, and are available from the Guangzhou Biobank Cohort Study Data Access Committee (gbcsdata@hku.hk) on reasonable request. (2) Mendelian randomization study: The GWAS summary statistics are publicly available through OpenGWAS or GWAS catalog.

## Abbreviations

SSB: Sugar sweetened beverages
PFJ: Pure fruit juice
BC: Breast cancer
MR: Mendelian randomization
GBCS: Guangzhou Biobank Cohort Study
GHHARE: Guangzhou Health and Happiness Association for the Respectable Elders
FFQ: Food Frequency Questionnaire
BMI: Body mass index
LDL-C: Low-density lipoprotein cholesterol
HDL-C: High-density lipoprotein cholesterol
HR: Hazard ratio
CI: Confidence interval
GWAS: Genome-wide association study
SNP: Single nucleotide polymorphism
IVW: Inverse-variance weighted
MR-PRESSO: MR pleiotropy residual sum and outlier
OR: Odds ratio
ER: Estrogen receptor
PUFA: Polyunsaturated fatty acid
TFA: Total fatty acid
