# Supplementary material for: The mediating roles of anthropo-metabolic biomarkers on the association between beverage consumption and breast cancer risk
Source: Nutr J. 2025 Mar 22;24:46. doi: 10.1186/s12937-025-01110-y (PMC11929343; doi:10.1186/s12937-025-01110-y)
Supplement: Supplementary file 1 — Additional file 1: Table S1. Beverage items used in the analysis. Table S2. Overview of genome-wide association study data. Table S3. Associations between beverage consumption and risk of breast cancer stratified by baseline age in the Guangzhou Biobank Cohort Study. Table S4. Associations between beverage consumption and risk of breast cancer stratified by baseline menopausal status in the Guangzhou Biobank Cohort Study. Table S5. Associations between beverage consumption and risk of breast cancer in 13,534 participants, excluding breast cancer or death events within the first year of follow-up in the Guangzhou Biobank Cohort Study. Table S6. Associations between beverage consumption and risk of breast cancer in the Guangzhou Biobank Cohort Study by proportional subdistribution hazards regression. Table S7. Associations between beverage consumption and risk of breast cancer in the Guangzhou Biobank Cohort Study using four partially adjusted models. Table S8. Associations between beverage consumption and 16 baseline anthropo-metabolic markers in the Guangzhou Biobank Cohort Study. Table S9. Mediation analysis with anthropo-metabolic markers as potential mediators for the association between beverage consumption and risk of breast cancer in the Guangzhou Biobank Cohort Study. Table S10. Summary information of the single nucleotide polymorphisms (SNPs) used as instrumental variables for beverage consumption in univariable Mendelian randomization. Table S11. Mendelian randomization estimates for the causal associations between sugar sweetened beverages consumption and estrogen receptor (ER)-positive/negative breast cancer. Table S12. Mendelian randomization estimates for the causal associations between sugar sweetened beverages consumption and anthropo-metabolic biomarkers. Table S13. Mendelian randomization estimates for the causal associations between anthropo-metabolic biomarkers and overall breast cancer. Figure S1. Flow diagram of participants selection. [file 12937_2025_1110_MOESM1_ESM.docx]

**Contents**

[**Table S1.** Beverage items used in the analysis **2**](#_Toc192153844)

[**Table S2.** Overview of genome-wide association study data **3**](#_Toc192153845)

[**Table S3.** Associations between beverage consumption and risk of breast cancer stratified by baseline age in the Guangzhou Biobank Cohort Study **5**](#_Toc192153846)

[**Table S4.** Associations between beverage consumption and risk of breast cancer stratified by baseline menopausal status in the Guangzhou Biobank Cohort Study **7**](#_Toc192153847)

[**Table S5.** Associations between beverage consumption and risk of breast cancer in 13,534 participants, excluding breast cancer or death events within the first year of follow-up in the Guangzhou Biobank Cohort Study **9**](#_Toc192153848)

[**Table S6.** Associations between beverage consumption and risk of breast cancer in the Guangzhou Biobank Cohort Study by proportional subdistribution hazards regression **10**](#_Toc192153849)

[**Table S7.** Associations between beverage consumption and risk of breast cancer in the Guangzhou Biobank Cohort Study using four partially adjusted models **11**](#_Toc192153850)

[**Table S8.** Associations between beverage consumption and 16 baseline anthropo-metabolic markers in the Guangzhou Biobank Cohort Study **13**](#_Toc192153851)

[**Table S9.** Mediation analysis with anthropo-metabolic markers as potential mediators for the association between beverage consumption and risk of breast cancer in the Guangzhou Biobank Cohort Study **15**](#_Toc192153852)

[**Table S10.** Summary information of the single nucleotide polymorphisms (SNPs) used as instrumental variables for beverage consumption in univariable Mendelian randomization **16**](#_Toc192153853)

[**Table S11.** Mendelian randomization estimates for the causal associations between sugar sweetened beverages consumption and estrogen receptor (ER)-positive/negative breast cancer **24**](#_Toc192153854)

[**Table S12.** Mendelian randomization estimates for the causal associations between sugar sweetened beverages consumption and anthropo-metabolic biomarkers **25**](#_Toc192153855)

[**Table S13.** Mendelian randomization estimates for the causal associations between anthropo-metabolic biomarkers and overall breast cancer **26**](#_Toc192153856)

[**Figure S1.** Flow diagram of participants selection **27**](#_Toc192153857)

# Table S1. Beverage items used in the analysis

| **No.** | **Beverage type** | **Beverage item** |
| --- | --- | --- |
|  | **Milk and alternatives** |  |
| 1 | Dairy-based milk | Whole milk; skim milk; flavored milk |
| 2 | Soy milk | Soy milk |
|  | **Sweet beverages** |  |
| 3 | Sugar sweetened beverages | Cola; Sprite; Vita; sweetened herbal beverage; sweetened boxed fruit juice |
| 4 | Pure fruit juice | Pure fruit juice |
|  | **Bitter beverages** |  |
| 5 | Coffee | Coffee |
| 6 | Tea | Green tea; black tea; oolong tea; ginseng tea; other tea |
| 7 | Alcoholic drinks | Wine; spirits; beer |

# Table S2. Overview of genome-wide association study data

| **Phenotype** | **Number of participants** | **Ancestry** | **Consortium**  **/Cohort** | **Pubmed**  **ID** |
| --- | --- | --- | --- | --- |
| **Exposures** |  |  |  |  |
| Dairy-based milk  consumption | 427,252 | European | UK Biobank | 32193382 |
| Soy milk  consumption | 31,889 | European | UK Biobank | 32193382 |
| Sugar sweetened  beverages consumption | 85,852 | European | UK Biobank | 31046077 |
| Pure fruit juice  consumption | 85,852 | European | UK Biobank | 31046077 |
| Coffee consumption | 335,909 | European | UK Biobank | 31046077 |
| Tea consumption | 335,812 | European | UK Biobank | 31046077 |
| Alcoholic drinks  consumption | 449,210 | European | UK Biobank | 32193382 |
| **Outcomes** |  |  |  |  |
| Breast cancer | 228,951 | European | Meta | 29059683 |
| ER-positive breast  cancer | 175,475 | European | Meta | 29059683 |
| ER-negative breast  cancer | 127,442 | European | Meta | 29059683 |
| **Potential mediators** |  |  |  |  |
| Body mass index | 322,154 | European | Meta | 25673413 |
| Waist circumference | 232,101 | European | Meta | 25673412 |
| Waist-to-hip ratio | 212,244 | European | Meta | 25673412 |
| Fasting glucose | 200,622 | European | Meta | 34059833 |
| Acetone | 130,311 | European (87.9%), East Asian, South Asian | Meta | 38448586 |
| Serum total  cholesterol | 136,016 | European (88.4%), East Asian, South Asian | Meta | 38448586 |
| LDL-C | 136,016 | European (88.4%), East Asian, South Asian | Meta | 38448586 |
| HDL-C | 136,016 | European (88.4%), East Asian, South Asian | Meta | 38448586 |
| Serum total  triglycerides | 136,016 | European (88.4%), East Asian, South Asian | Meta | 38448586 |
| Total fatty acids | 136,016 | European (88.4%), East Asian, South Asian | Meta | 38448586 |
| Monounsaturated fatty acids | 136,016 | European (88.4%), East Asian, South Asian | Meta | 38448586 |
| Ratio of  monounsaturated fatty acids to TFAs | 136,016 | European (88.4%), East Asian, South Asian | Meta | 38448586 |
| Polyunsaturated fatty acids | 136,016 | European (88.4%), East Asian, South Asian | Meta | 38448586 |
| Ratio of  polyunsaturated fatty acids to TFAs | 136,016 | European (88.4%), East Asian, South Asian | Meta | 38448586 |
| Ratio of omega-3  fatty acids to TFAs | 136,016 | European (88.4%), East Asian, South Asian | Meta | 38448586 |
| Ratio of omega-6  fatty acids to TFAs | 126,671 | European (87.5%), East Asian, South Asian | Meta | 38448586 |
| Bilirubin | 56,577 | European | BioVU | 33441150 |
| Urea | 8,238 | European | CLSA cohort | 36635386 |
| Creatinine | 8,239 | European | CLSA cohort | 36635386 |
| Urate | 110,347 | European | Meta | 23263486 |
| Valine | 8,247 | European | CLSA cohort | 36635386 |
| Lysine | 8,250 | European | CLSA cohort | 36635386 |
| Arginine | 8,237 | European | CLSA cohort | 36635386 |
| Glutamine | 8,253 | European | CLSA cohort | 36635386 |
| Creatine | 8,285 | European | CLSA cohort | 36635386 |

Abbreviation: ER, estrogen receptor; LDL-C, low-density lipoprotein cholesterol; HDL-C, high-density lipoprotein cholesterol; TFAs, total fatty acids; BioVU, Vanderbilt University Medical Center's biobank; CLSA, Canadian Longitudinal Study on Aging.

# Table S3. Associations between beverage consumption and risk of breast cancer stratified by baseline age in the Guangzhou Biobank Cohort Study

|  | **Age<60 years** | | |  | **Age≥60 years** | | |  |
| --- | --- | --- | --- | --- | --- | --- | --- | --- |
|  | **N** | **Adjusted HR (95% CI) ^a^** | ***P* ^b^** |  | **N** | **Adjusted HR (95% CI) ^a^** | ***P* ^b^** | ***P-*interaction** |
| **Dairy-based milk** |  |  |  |  |  |  |  | 0.73 |
| <1 portion/week | 3,929 | 1.00 |  |  | 5,444 | 1.00 |  |  |
| 1-2 portions/week | 651 | 1.39 (0.82, 2.38) | 0.22 |  | 537 | 0.86 (0.41, 1.78) | 0.68 |  |
| 3-6 portions/week | 802 | 1.07 (0.62, 1.83) | 0.80 |  | 695 | 0.89 (0.47, 1.68) | 0.72 |  |
| >6 portions/week | 625 | 1.48 (0.87, 2.51) | 0.15 |  | 884 | 1.37 (0.84, 2.25) | 0.21 |  |
| per 1 portion |  | 1.05 (0.98, 1.12) | 0.20 |  |  | 1.03 (0.96, 1.09) | 0.41 |  |
| **Soy milk** |  |  |  |  |  |  |  | 0.90 |
| <1 portion/week | 5,002 | 1.00 |  |  | 6,516 | 1.00 |  |  |
| 1-2 portions/week | 700 | 1.10 (0.64, 1.86) | 0.73 |  | 683 | 0.97 (0.53, 1.77) | 0.91 |  |
| 3-6 portions/week | 234 | 0.20 (0.03, 1.46) | 0.11 |  | 262 | 0.44 (0.11, 1.79) | 0.25 |  |
| >6 portions/week | 71 | 0.65 (0.09, 4.67) | 0.67 |  | 99 | 0.60 (0.08, 4.31) | 0.61 |  |
| per 1 portion |  | 0.88 (0.72, 1.08) | 0.23 |  |  | 0.88 (0.71, 1.09) | 0.23 |  |
| **Sugar sweetened beverages** | |  |  |  |  |  |  | 0.049 |
| <1 portion/week | 5,123 | 1.00 |  |  | 6,948 | 1.00 |  |  |
| ≥1 portion/week | 884 | 2.01 (1.33, 3.04) | 0.001 |  | 612 | 1.01 (0.52, 1.95) | 0.98 |  |
| per 1 portion |  | 1.07 (1.05, 1.09) | <0.001 |  |  | 0.85 (0.61, 1.18) | 0.33 |  |
| **Pure fruit juice** |  |  |  |  |  |  |  | 0.34 |
| <1 portion/week | 5,818 | 1.00 |  |  | 7,406 | 1.00 |  |  |
| ≥1 portion/week | 189 | 1.58 (0.69, 3.59) | 0.28 |  | 154 | 0.76 (0.19, 3.09) | 0.70 |  |
| per 1 portion |  | 1.19 (0.94, 1.50) | 0.15 |  |  | 1.09 (0.81, 1.47) | 0.58 |  |
| **Coffee** |  |  |  |  |  |  |  | 0.77 |
| <1 portion/week | 5,839 | 1.00 |  |  | 7,443 | 1.00 |  |  |
| ≥1 portion/week | 168 | 1.08 (0.40, 2.94) | 0.88 |  | 117 | 1.43 (0.45, 4.54) | 0.55 |  |
| per 1 portion |  | 0.80 (0.41, 1.53) | 0.49 |  |  | 0.86 (0.45, 1.64) | 0.65 |  |
| **Tea** |  |  |  |  |  |  |  | 0.77 |
| <1 portion/week | 4,458 | 1.00 |  |  | 5,329 | 1.00 |  |  |
| ≥1 portion/week | 1549 | 1.06 (0.71, 1.60) | 0.78 |  | 2,231 | 0.97 (0.65, 1.44) | 0.89 |  |
| per 1 portion |  | 1.01 (0.99, 1.02) | 0.57 |  |  | 1.00 (0.99, 1.02) | 0.89 |  |
| **Alcoholic drinks** |  |  |  |  |  |  |  | 0.19 |
| <1 portion/week | 5,833 | 1.00 |  |  | 7,448 | 1.00 |  |  |
| ≥1 portion/week | 174 | 1.54 (0.66, 3.59) | 0.31 |  | 112 | 0.52 (0.07, 3.79) | 0.52 |  |
| per 1 portion |  | 1.12 (0.65, 1.92) | 0.68 |  |  | 0.83 (0.28, 2.46) | 0.74 |  |

Abbreviations: HR, hazard ratio; CI, confidence interval. 1 portion= 250 milliliters

^a^ Adjusted for age, education level, occupation, annual personal income, smoking status, alcohol use, physical activity, age at menarche and menopause, parity and breastfeeding history, oral contraceptive use, hormone replacement therapy, self-reported health status, family history of breast cancer, and daily dietary energy intake.

^b^ *P*-value was for the adjusted model.

# Table S4. Associations between beverage consumption and risk of breast cancer stratified by baseline menopausal status in the Guangzhou Biobank Cohort Study

|  | **Premenopausal** | | |  | **Postmenopausal** | | |  |
| --- | --- | --- | --- | --- | --- | --- | --- | --- |
|  | **N** | **Adjusted HR (95% CI) ^a^** | ***P* ^b^** |  | **N** | **Adjusted HR (95% CI) ^a^** | ***P* ^b^** | ***P-*interaction** |
| **Dairy-based milk** |  |  |  |  |  |  |  | 0.93 |
| <1 portion/week | 366 | 1.00 |  |  | 9,007 | 1.00 |  |  |
| 1-2 portions/week | 79 | 1.02 (0.21, 4.89) | 0.98 |  | 1,109 | 1.16 (0.74, 1.81) | 0.51 |  |
| 3-6 portions/week | 91 | 1.15 (0.30, 4.44) | 0.84 |  | 1,406 | 0.96 (0.63, 1.48) | 0.87 |  |
| >6 portions/week | 59 | 1.79 (0.44, 7.19) | 0.41 |  | 1,450 | 1.39 (0.96, 2.02) | 0.08 |  |
| per 1 portion |  | 1.07 (0.89, 1.30) | 0.46 |  |  | 1.03 (0.98, 1.08) | 0.20 |  |
| **Soy milk** |  |  |  |  |  |  |  | 0.54 |
| <1 portion/week | 486 | 1.00 |  |  | 11,032 | 1.00 |  |  |
| 1-2 portions/week | 79 | 0.16 (0.02, 1.41) | 0.10 |  | 1,304 | 1.11 (0.74, 1.67) | 0.61 |  |
| 3-6 portions/week | 19 | 0.00 (0.00, Inf) | >0.99 |  | 477 | 0.34 (0.11, 1.07) | 0.06 |  |
| >6 portions/week | 11 | 0.00 (0.00, Inf) | >0.99 |  | 159 | 0.71 (0.18, 2.87) | 0.63 |  |
| per 1 portion |  | 0.20 (0.03, 1.55) | 0.12 |  |  | 0.90 (0.78, 1.04) | 0.17 |  |
| **Sugar sweetened beverages** | |  |  |  |  |  |  | 0.79 |
| <1 portion/week | 508 | 1.00 |  |  | 11,563 | 1.00 |  |  |
| ≥1 portion/week | 87 | 1.77 (0.55, 5.65) | 0.34 |  | 1,409 | 1.55 (1.08, 2.23) | 0.02 |  |
| per 1 portion |  | 1.00 (0.79, 1.28) | 0.99 |  |  | 1.05 (1.03, 1.07) | <0.001 |  |
| **Pure fruit juice** |  |  |  |  |  |  |  | 0.87 |
| <1 portion/week | 570 | 1.00 |  |  | 12,654 | 1.00 |  |  |
| ≥1 portion/week | 25 | 1.27 (0.16, 10.19) | 0.82 |  | 318 | 1.20 (0.57, 2.56) | 0.63 |  |
| per 1 portion |  | 1.10 (0.46, 2.61) | 0.83 |  |  | 1.14 (0.94, 1.37) | 0.18 |  |
| **Coffee** |  |  |  |  |  |  |  | 0.25 |
| <1 portion/week | 567 | 1.00 |  |  | 12,715 | 1.00 |  |  |
| ≥1 portion/week | 28 | 1.77 (0.34, 9.31) | 0.50 |  | 257 | 0.97 (0.40, 2.37) | 0.95 |  |
| per 1 portion |  | 0.95 (0.37, 2.42) | 0.91 |  |  | 0.75 (0.42, 1.34) | 0.34 |  |
| **Tea** |  |  |  |  |  |  |  | 0.14 |
| <1 portion/week | 442 | 1.00 |  |  | 9,345 | 1.00 |  |  |
| ≥1 portion/week | 153 | 1.58 (0.56, 4.44) | 0.38 |  | 3,627 | 0.97 (0.72, 1.30) | 0.83 |  |
| per 1 portion |  | 1.02 (0.99, 1.06) | 0.23 |  |  | 1.00 (0.99, 1.01) | 0.89 |  |
| **Alcoholic drinks** |  |  |  |  |  |  |  | 0.93 |
| <1 portion/week | 568 | 1.00 |  |  | 12,713 | 1.00 |  |  |
| ≥1 portion/week | 27 | 1.20 (0.14, 10.46) | 0.87 |  | 259 | 1.23 (0.54, 2.81) | 0.63 |  |
| per 1 portion |  | 1.19 (0.20, 6.96) | 0.85 |  |  | 0.99 (0.74, 1.32) | 0.96 |  |

Abbreviations: HR, hazard ratio; CI, confidence interval. 1 portion= 250 milliliters

^a^ Adjusted for age, education level, occupation, annual personal income, smoking status, alcohol use, physical activity, age at menarche and menopause, parity and breastfeeding history, oral contraceptive use, hormone replacement therapy, self-reported health status, family history of breast cancer, and daily dietary energy intake.

^b^ *P*-value was for the adjusted model.

# Table S5. Associations between beverage consumption and risk of breast cancer in 13,534 participants, excluding breast cancer or death events within the first year of follow-up in the Guangzhou Biobank Cohort Study

|  | **N** | **Crude HR (95% CI)** | **Adjusted HR (95% CI) ^a^** | ***P* ^b^** |
| --- | --- | --- | --- | --- |
| **Dairy-based milk** |  |  |  |  |
| <1 portion/week | 9,350 | 1.00 | 1.00 |  |
| 1-2 portions/week | 1,185 | 1.26 (0.81, 1.96) | 1.08 (0.70, 1.69) | 0.72 |
| 3-6 portions/week | 1,496 | 1.16 (0.77, 1.74) | 0.97 (0.64, 1.47) | 0.87 |
| >6 portions/week | 1,503 | 1.64 (1.15, 2.34) | 1.41 (0.98, 2.04) | 0.06 |
| per 1 portion |  | 1.05 (1.01, 1.09) | 1.03 (0.99, 1.08) | 0.16 |
| **Soy milk** |  |  |  |  |
| <1 portion/week | 11,489 | 1.00 | 1.00 |  |
| 1-2 portions/week | 1,380 | 1.10 (0.73, 1.64) | 1.03 (0.69, 1.54) | 0.89 |
| 3-6 portions/week | 495 | 0.33 (0.11, 1.03) | 0.32 (0.10, 1.01) | 0.05 |
| >6 portions/week | 170 | 0.67 (0.17, 2.69) | 0.65 (0.16, 2.63) | 0.55 |
| per 1 portion |  | 0.90 (0.77, 1.04) | 0.88 (0.76, 1.03) | 0.10 |
| **Sugar sweetened beverages** | |  |  |  |
| <1 portion/week | 12,039 | 1.00 | 1.00 |  |
| ≥1 portion/week | 1,495 | 1.70 (1.21, 2.38) | 1.58 (1.12, 2.24) | 0.01 |
| per 1 portion |  | 1.05 (1.03, 1.07) | 1.05 (1.03, 1.07) | <0.001 |
| **Pure fruit juice** |  |  |  |  |
| <1 portion/week | 13,191 | 1.00 | 1.00 |  |
| ≥1 portion/week | 343 | 1.36 (0.67, 2.76) | 1.26 (0.62, 2.56) | 0.52 |
| per 1 portion |  | 1.14 (0.96, 1.35) | 1.14 (0.95, 1.37) | 0.15 |
| **Coffee** |  |  |  |  |
| <1 portion/week | 13,250 | 1.00 | 1.00 |  |
| ≥1 portion/week | 284 | 1.48 (0.70, 3.14) | 1.23 (0.57, 2.62) | 0.60 |
| per 1 portion |  | 0.88 (0.57, 1.36) | 0.83 (0.52, 1.31) | 0.41 |
| **Tea** |  |  |  |  |
| <1 portion/week | 9,761 | 1.00 | 1.00 |  |
| ≥1 portion/week | 3,773 | 0.99 (0.74, 1.32) | 1.03 (0.77, 1.38) | 0.82 |
| per 1 portion |  | 1.00 (0.99, 1.01) | 1.00 (0.99, 1.02) | 0.51 |
| **Alcoholic drinks** |  |  |  |  |
| <1 portion/week | 13,248 | 1.00 | 1.00 |  |
| ≥1 portion/week | 286 | 1.48 (0.70, 3.13) | 1.24 (0.57, 2.67) | 0.59 |
| per 1 portion |  | 1.00 (0.83, 1.21) | 0.99 (0.75, 1.31) | 0.95 |

Abbreviations: HR, hazard ratio; CI, confidence interval. 1 portion= 250 milliliters

^a^ Adjusted for age, education level, occupation, annual personal income, smoking status, alcohol use, physical activity, age at menarche and menopause, parity and breastfeeding history, oral contraceptive use, hormone replacement therapy, self-reported health status, family history of breast cancer, and daily dietary energy intake.

^b^ *P*-value was for the adjusted model.

# Table S6. Associations between beverage consumption and risk of breast cancer in the Guangzhou Biobank Cohort Study by proportional subdistribution hazards regression

|  | **Subdistribution HR (95% CI) ^a^** | ***P*** |
| --- | --- | --- |
| **Dairy-based milk** |  |  |
| <1 portion/week | 1.00 |  |
| 1-2 portions/week | 1.15 (0.75, 1.75) | 0.52 |
| 3-6 portions/week | 0.98 (0.65, 1.48) | 0.93 |
| >6 portions/week | 1.40 (0.98, 1.99) | 0.06 |
| per 1 portion | 1.03 (0.99, 1.08) | 0.12 |
| **Soy milk** |  |  |
| <1 portion/week | 1.00 |  |
| 1-2 portions/week | 1.04 (0.70, 1.55) | 0.85 |
| 3-6 portions/week | 0.32 (0.10, 0.99) | 0.048 |
| >6 portions/week | 0.62 (0.15, 2.47) | 0.49 |
| per 1 portion | 0.88 (0.76, 1.02) | 0.08 |
| **Sugar sweetened beverages** | |  |
| <1 portion/week | 1.00 |  |
| ≥1 portion/week | 1.58 (1.12, 2.22) | 0.009 |
| per 1 portion | 1.05 (1.03, 1.07) | <0.001 |
| **Pure fruit juice** |  |  |
| <1 portion/week | 1.00 |  |
| ≥1 portion/week | 1.23 (0.61, 2.51) | 0.56 |
| per 1 portion | 1.13 (0.95, 1.35) | 0.16 |
| **Coffee** |  |  |
| <1 portion/week | 1.00 |  |
| ≥1 portion/week | 1.18 (0.56, 2.52) | 0.66 |
| per 1 portion | 0.82 (0.62, 1.08) | 0.15 |
| **Tea** |  |  |
| <1 portion/week | 1.00 |  |
| ≥1 portion/week | 1.02 (0.77, 1.36) | 0.89 |
| per 1 portion | 1.00 (0.99, 1.01) | 0.64 |
| **Alcoholic drinks** |  |  |
| <1 portion/week | 1.00 |  |
| ≥1 portion/week | 1.21 (0.57, 2.57) | 0.63 |
| per 1 portion | 0.99 (0.83, 1.17) | 0.87 |

Abbreviations: HR, hazard ratio; CI, confidence interval. 1 portion= 250 milliliters

^a^ Adjusted for age, education level, occupation, annual personal income, smoking status, alcohol use, physical activity, age at menarche and menopause, parity and breastfeeding history, oral contraceptive use, hormone replacement therapy, self-reported health status, family history of breast cancer, and daily dietary energy intake.

# Table S7. Associations between beverage consumption and risk of breast cancer in the Guangzhou Biobank Cohort Study using four partially adjusted models

|  | **Model 1** | |  | **Model 2** | |  | **Model 3** | |  | **Model 4** | |
| --- | --- | --- | --- | --- | --- | --- | --- | --- | --- | --- | --- |
|  | **Adjusted HR (95% CI)** | **P** |  | **Adjusted HR (95% CI)** | ***P*** |  | **Adjusted HR (95% CI)** | **P** |  | **Adjusted HR (95% CI)** | ***P*** |
| **Milk** |  |  |  |  |  |  |  |  |  |  |  |
| <1 portion/week | 1.00 |  |  | 1.00 |  |  | 1.00 |  |  | 1.00 |  |
| 1-2 portions/week | 1.18 (0.77, 1.81) | 0.44 |  | 1.26 (0.82, 1.93) | 0.28 |  | 1.27 (0.83, 1.94) | 0.27 |  | 1.29 (0.84, 1.97) | 0.24 |
| 3-6 portions/week | 1.01 (0.67, 1.52) | 0.96 |  | 1.11 (0.74, 1.67) | 0.61 |  | 1.12 (0.74, 1.68) | 0.59 |  | 1.13 (0.75, 1.70) | 0.55 |
| >6 portions/week | 1.44 (1.01, 2.07) | 0.046 |  | 1.61 (1.13, 2.30) | 0.009 |  | 1.61 (1.12, 2.29) | 0.009 |  | 1.62 (1.13, 2.31) | 0.008 |
| per 1 portion | 1.04 (0.99, 1.08) | 0.12 |  | 1.05 (1.01, 1.09) | 0.02 |  | 1.05 (1.01, 1.09) | 0.02 |  | 1.05 (1.01, 1.09) | 0.02 |
| **Soy milk** |  |  |  |  |  |  |  |  |  |  |  |
| <1 portion/week | 1.00 |  |  | 1.00 |  |  | 1.00 |  |  | 1.00 |  |
| 1-2 portions/week | 1.01 (0.68, 1.50) | 0.97 |  | 1.09 (0.73, 1.62) | 0.67 |  | 1.08 (0.73, 1.60) | 0.70 |  | 1.07 (0.72, 1.60) | 0.72 |
| 3-6 portions/week | 0.30 (0.10, 0.94) | 0.04 |  | 0.33 (0.10, 1.03) | 0.06 |  | 0.31 (0.10, 0.98) | 0.046 |  | 0.31 (0.10, 0.97) | 0.04 |
| >6 portions/week | 0.60 (0.15, 2.41) | 0.47 |  | 0.67 (0.17, 2.72) | 0.58 |  | 0.65 (0.16, 2.61) | 0.54 |  | 0.64 (0.16, 2.58) | 0.53 |
| per 1 portion | 0.87 (0.75, 1.01) | 0.07 |  | 0.89 (0.77, 1.03) | 0.13 |  | 0.89 (0.77, 1.02) | 0.10 |  | 0.88 (0.76, 1.02) | 0.10 |
| **Sugar sweetened beverage** |  |  |  |  |  |  |  |  |  |  |  |
| <1 portion/week | 1.00 |  |  | 1.00 |  |  | 1.00 |  |  | 1.00 |  |
| ≥1 portion/week | 1.60 (1.14, 2.25) | 0.007 |  | 1.58 (1.12, 2.22) | 0.009 |  | 1.59 (1.13, 2.24) | 0.008 |  | 1.67 (1.19, 2.35) | 0.003 |
| per 1 portion | 1.05 (1.02, 1.07) | <0.001 |  | 1.05 (1.02, 1.07) | <0.001 |  | 1.05 (1.02, 1.07) | <0.001 |  | 1.05 (1.03, 1.07) | <0.001 |
| **Pure fruit juice** |  |  |  |  |  |  |  |  |  |  |  |
| <1 portion/week | 1.00 |  |  | 1.00 |  |  | 1.00 |  |  | 1.00 |  |
| ≥1 portion/week | 1.21 (0.60, 2.46) | 0.59 |  | 1.27 (0.63, 2.57) | 0.51 |  | 1.28 (0.63, 2.59) | 0.50 |  | 1.29 (0.63, 2.61) | 0.49 |
| per 1 portion | 1.12 (0.94, 1.34) | 0.20 |  | 1.13 (0.95, 1.34) | 0.17 |  | 1.14 (0.95, 1.36) | 0.15 |  | 1.13 (0.95, 1.35) | 0.16 |
| **Coffee** |  |  |  |  |  |  |  |  |  |  |  |
| <1 portion/week | 1.00 |  |  | 1.00 |  |  | 1.00 |  |  | 1.00 |  |
| ≥1 portion/week | 1.18 (0.55, 2.51) | 0.67 |  | 1.36 (0.64, 2.90) | 0.42 |  | 1.33 (0.63, 2.83) | 0.45 |  | 1.39 (0.65, 2.96) | 0.39 |
| per 1 portion | 0.82 (0.52, 1.29) | 0.39 |  | 0.86 (0.55, 1.34) | 0.50 |  | 0.85 (0.55, 1.33) | 0.48 |  | 0.86 (0.56, 1.34) | 0.52 |
| **Tea** |  |  |  |  |  |  |  |  |  |  |  |
| <1 portion/week | 1.00 |  |  | 1.00 |  |  | 1.00 |  |  | 1.00 |  |
| ≥1 portion/week | 0.99 (0.75, 1.32) | 0.96 |  | 1.02 (0.77, 1.36) | 0.87 |  | 1.00 (0.75, 1.32) | 0.98 |  | 0.99 (0.74, 1.31) | 0.93 |
| per 1 portion | 1.00 (0.99, 1.01) | 0.85 |  | 1.00 (0.99, 1.01) | 0.77 |  | 1.00 (0.99, 1.01) | 0.99 |  | 1.00 (0.99, 1.01) | 0.95 |
| **Alcoholic beverage** |  |  |  |  |  |  |  |  |  |  |  |
| <1 portion/week | 1.00 |  |  | 1.00 |  |  | 1.00 |  |  | 1.00 |  |
| ≥1 portion/week | 1.19 (0.56, 2.53) | 0.66 |  | 1.38 (0.64, 2.96) | 0.41 |  | 1.32 (0.62, 2.82) | 0.47 |  | 1.40 (0.66, 2.97) | 0.39 |
| per 1 portion | 0.98 (0.72, 1.34) | 0.92 |  | 1,00 (0.81, 1.24) | 0.99 |  | 0.99 (0.78, 1.25) | 0.94 |  | 1.00 (0.81, 1.23) | >0.99 |

Abbreviations: HR, hazard ratio; CI, confidence interval. 1 portion= 250 milliliters

Model 1 was adjusted for age, education level, occupation, annual personal income, and daily dietary energy intake; Model 2 was adjusted for age, smoking status, alcohol use, physical activity, and daily dietary energy intake; Model 3 was adjusted for age, age at menarche and menopause, parity and breastfeeding history, and daily dietary energy intake; Model 4 was adjusted for age, oral contraceptive use, hormone replacement therapy, self-reported health status, family history of breast cancer, and daily dietary energy intake.

# Table S8. Associations between beverage consumption and 16 baseline anthropo-metabolic markers in the Guangzhou Biobank Cohort Study

| **Exposure** | **Mediator** | **Crude β (95% CI)** | **Adjusted β (95% CI) ^a^** | ***P* ^b^** |
| --- | --- | --- | --- | --- |
| **Soy milk** (3-6 portions/week vs. <1 portion/week) | Body mass index, kg/m^2^ | 0.49 (0.19, 0.80) | 0.58 (0.27, 0.88) | <0.001 |
|  | Waist circumference, cm | 0.85 (0.07, 1.62) | 1.15 (0.39, 1.91) | 0.003 |
|  | Waist-to-hip ratio | 0.00 (-0.01, 0.00) | 0.00 (0.00, 0.01) | 0.73 |
|  | Fasting glucose, mmol/L | -0.05 (-0.21, 0.11) | -0.01 (-0.17, 0.14) | 0.86 |
|  | Serum total cholesterol, mmol/L | -0.06 (-0.17, 0.05) | -0.05 (-0.15, 0.06) | 0.36 |
|  | LDL-C, mmol/L | -0.06 (-0.12, 0.00) | -0.04 (-0.10, 0.02) | 0.18 |
|  | HDL-C, mmol/L | -0.02 (-0.06, 0.01) | -0.03 (-0.06, 0.01) | 0.15 |
|  | Serum total triglycerides, mmol/L | -0.06 (-0.17, 0.05) | -0.04 (-0.15, 0.08) | 0.54 |
|  | Bilirubin, μmol/L | -0.01 (-0.43, 0.41) | -0.05 (-0.47, 0.37) | 0.81 |
|  | Blood urea nitrogen, mmol/L | -0.16 (-0.34, 0.01) | -0.11 (-0.28, 0.06) | 0.22 |
|  | Creatinine, μmol/L | -1.55 (-3.60, 0.50) | -0.73 (-2.77, 1.30) | 0.48 |
|  | Uric acid, μmol/L | 0.48 (-8.55, 9.52) | 1.53 (-7.47, 10.53) | 0.74 |
| **Sugar sweetened beverages** (≥1 portion/week vs. <1 portion/week) | Body mass index, kg/m^2^ | 0.24 (0.05, 0.42) | 0.23 (0.05, 0.41) | 0.01 |
|  | Waist circumference, cm | -0.64 (-1.10, -0.17) | 0.11 (-0.35, 0.56) | 0.65 |
|  | Waist-to-hip ratio | 0.00 (-0.01, 0.00) | 0.00 (0.00, 0.01) | 0.24 |
|  | Fasting glucose, mmol/L | -0.32 (-0.42, -0.23) | -0.16 (-0.26, -0.07) | <0.001 |
|  | Serum total cholesterol, mmol/L | 0.03 (-0.03, 0.09) | 0.01 (-0.06, 0.07) | 0.82 |
|  | LDL-C, mmol/L | 0.09 (0.05, 0.13) | 0.04 (0.01, 0.08) | 0.02 |
|  | HDL-C, mmol/L | -0.01 (-0.04, 0.01) | -0.01 (-0.03, 0.01) | 0.37 |
|  | Serum total triglycerides, mmol/L | 0.03 (-0.04, 0.10) | 0.05 (-0.02, 0.12) | 0.17 |
|  | Bilirubin, μmol/L | -0.08 (-0.53, 0.37) | -0.13 (-0.59, 0.32) | 0.56 |
|  | Blood urea nitrogen, mmol/L | -0.11 (-0.30, 0.08) | -0.07 (-0.25, 0.12) | 0.49 |
|  | Creatinine, μmol/L | -1.14 (-3.36, 1.08) | -0.53 (-2.73, 1.66) | 0.63 |
|  | Uric acid, μmol/L | 12.35 (2.59, 22.12) | 14.68 (4.98, 24.37) | 0.003 |

Abbreviations: CI, confidence interval. 1 portion= 250 milliliters; LDL-C, low-density lipoprotein cholesterol; HDL-C, high-density lipoprotein cholesterol.

^a^ Adjusted for age, education level, occupation, annual personal income, smoking status, alcohol use, physical activity, age at menarche and menopause, parity and breastfeeding history, oral contraceptive use, hormone replacement therapy, self-reported health status, family history of breast cancer, and daily dietary energy intake.

^b^ *P*-value was for the adjusted model.

# Table S9. Mediation analysis with anthropo-metabolic markers as potential mediators for the association between beverage consumption and risk of breast cancer in the Guangzhou Biobank Cohort Study

| **Exposures** | **Mediator** | **HR (95% CI) ^a^** | | **Proportion mediated (%) (95% CI)** | ***P* ^b^** |
| --- | --- | --- | --- | --- | --- |
|  |  | **Unadjusted for**  **potential mediator(s)** | **Adjusted for**  **potential mediator(s)** |  |  |
| **Soy milk** (3-6 portions/week vs. <1 portion/week) | Body mass index, kg/m^2^ | 0.31 (0.10, 0.98) | 0.31 (0.10, 0.97) | - | - |
|  | Waist circumference, cm |  | 0.31 (0.10, 0.97) | - | - |
| **Sugar sweetened beverages** (≥1 portion/week vs. <1 portion/week) | Body mass index, kg/m^2^ | 1.05 (1.03, 1.07) | 1.56 (1.10, 2.20) | 4.2 (0.9-17.1) | 0.007 |
|  | Fasting glucose, mmol/L |  | 1.60 (1.13, 2.26) | - | - |
|  | LDL-C, mmol/L |  | 1.58 (1.12, 2.24) | - | - |
|  | Uric acid, μmol/L |  | 1.48 (0.68, 3.20) | 18.8 (1.5-77.5) | 0.02 |

Abbreviations: HR, hazard ratio; CI, confidence interval; LDL-C, low-density lipoprotein cholesterol. 1 portion= 250 milliliters.

^a^ Adjusted for age, education level, occupation, annual personal income, smoking status, alcohol use, physical activity, age at menarche and menopause, parity and breastfeeding history, oral contraceptive use, hormone replacement therapy, self-reported health status, family history of breast cancer, and daily dietary energy intake.

^b^ Since the transform that the SAS “mediate” macro used to get the confidence interval for mediation proportion does not go below zero, the confidence interval does not contain the null value (0) even there is a nonsignificant *P*-value. According to the guidance of the “mediate” macro, we need to pay attention to the *P*-value.

# Table S10. Summary information of the single nucleotide polymorphisms (SNPs) used as instrumental variables for beverage consumption in univariable Mendelian randomization

| **SNP** | **Effect allele** | **Other allele** | **β** | **EAF** | **SE** | ***P*** |
| --- | --- | --- | --- | --- | --- | --- |
| **Dairy-based milk** | |  |  |  |  |  |
| rs11075922 | C | T | 0.0052873 | 0.611034 | 0.0011542 | 4.60E-06 |
| rs11083174 | C | T | 0.006241 | 0.735774 | 0.0013033 | 1.70E-06 |
| rs113690218 | G | T | -0.0078957 | 0.875773 | 0.0017275 | 4.90E-06 |
| rs115853505 | C | T | 0.0116033 | 0.942166 | 0.0024764 | 2.80E-06 |
| rs142924214 | A | T | 0.0241674 | 0.986955 | 0.0051125 | 2.30E-06 |
| rs151207429 | C | A | 0.0263728 | 0.988176 | 0.0055491 | 2.00E-06 |
| rs2964030 | A | C | -0.0083008 | 0.827452 | 0.0014926 | 2.70E-08 |
| rs34045187 | C | T | 0.006949 | 0.713753 | 0.0013053 | 1.00E-07 |
| rs34275555 | C | T | -0.0058927 | 0.293729 | 0.0012765 | 3.90E-06 |
| rs377203031 | C | T | 0.0068057 | 0.798157 | 0.0014616 | 3.20E-06 |
| rs550497 | C | T | 0.0052702 | 0.446921 | 0.0011413 | 3.90E-06 |
| rs55679746 | C | G | 0.0068276 | 0.792735 | 0.0014015 | 1.10E-06 |
| rs57412027 | A | T | 0.0060551 | 0.754194 | 0.0013141 | 4.10E-06 |
| rs6754311 | T | C | 0.0087194 | 0.743232 | 0.001345 | 9.10E-11 |
| rs7013121 | C | A | -0.0070037 | 0.789007 | 0.0013825 | 4.10E-07 |
| rs7016572 | C | T | 0.0080064 | 0.860906 | 0.0016325 | 9.40E-07 |
| rs72731943 | A | G | -0.0115829 | 0.919268 | 0.0021015 | 3.60E-08 |
| rs74401181 | C | T | -0.0144545 | 0.965021 | 0.0031592 | 4.80E-06 |
| rs8050320 | G | A | 0.0087188 | 0.901086 | 0.0018898 | 4.00E-06 |
| rs9542208 | T | C | -0.0132908 | 0.959798 | 0.0028725 | 3.70E-06 |
|  |  |  |  |  |  |  |
| **Soy milk** |  |  |  |  |  |  |
| rs10008010 | A | C | -0.0423899 | 0.569492 | 0.0091972 | 4.20E-06 |
| rs10488830 | A | G | -0.0793989 | 0.919823 | 0.0168921 | 2.10E-06 |
| rs12470316 | C | T | 0.04873 | 0.699531 | 0.0099717 | 1.20E-06 |
| rs12782575 | G | A | 0.0539993 | 0.808303 | 0.0116478 | 4.30E-06 |
| rs143870099 | G | A | -0.161606 | 0.981448 | 0.0350695 | 3.80E-06 |
| rs1509555 | A | T | -0.0711398 | 0.887638 | 0.0145121 | 7.70E-07 |
| rs17416088 | C | T | -0.0589959 | 0.845036 | 0.0126602 | 2.80E-06 |
| rs1882914 | T | A | -0.0547163 | 0.17876 | 0.0119337 | 3.60E-06 |
| rs1896634 | T | C | -0.0595842 | 0.790286 | 0.0112473 | 1.10E-07 |
| rs3130662 | C | A | 0.0562186 | 0.814889 | 0.0117467 | 1.80E-06 |
| rs35656416 | C | A | 0.0672209 | 0.875921 | 0.0141502 | 2.10E-06 |
| rs35657069 | G | A | 0.109735 | 0.96146 | 0.0236928 | 4.00E-06 |
| rs55957544 | T | C | -0.0794832 | 0.917824 | 0.0165916 | 1.40E-06 |
| rs56127503 | T | C | 0.054974 | 0.821755 | 0.011921 | 4.60E-06 |
| rs56221912 | G | C | 0.0947546 | 0.93614 | 0.0187968 | 4.20E-07 |
| rs6776772 | C | T | 0.0435416 | 0.452216 | 0.0093102 | 3.00E-06 |
| rs73487126 | C | G | -0.130166 | 0.968647 | 0.0263202 | 7.10E-07 |
| rs7396314 | T | G | -0.0441733 | 0.627767 | 0.0096315 | 4.30E-06 |
| rs7417538 | G | A | 0.0465171 | 0.642057 | 0.0095535 | 1.50E-06 |
| rs74847990 | C | T | 0.210958 | 0.988758 | 0.0433347 | 1.20E-06 |
| rs75132027 | T | C | -0.188367 | 0.985762 | 0.0401436 | 3.40E-06 |
| rs76936221 | G | A | 0.0773033 | 0.90087 | 0.0153264 | 4.40E-07 |
| rs7810473 | A | G | -0.0438206 | 0.581757 | 0.0092616 | 2.50E-06 |
| rs78202195 | C | T | -0.0950177 | 0.94439 | 0.0201578 | 3.00E-06 |
| rs9545184 | T | A | -0.185716 | 0.9865 | 0.039755 | 3.50E-06 |
| rs9660298 | A | G | 0.0457411 | 0.468308 | 0.0092621 | 8.60E-07 |
| rs9687441 | G | A | -0.0528417 | 0.565467 | 0.0092526 | 1.40E-08 |
|  |  |  |  |  |  |  |
| **Sugar sweetened beverage** | | |  |  |  |  |
| rs13114522 | A | C | -0.0256351 | 0.981373 | 0.0055073 | 3.25E-06 |
| rs16970214 | T | C | -0.0071498 | 0.56782 | 0.0014662 | 1.08E-06 |
| rs2472297 | C | T | 0.008542 | 0.736116 | 0.0016435 | 2.02E-07 |
| rs304216 | C | G | -0.0074541 | 0.278588 | 0.0016142 | 3.88E-06 |
| rs55872725 | C | T | 0.0095311 | 0.59479 | 0.0014826 | 1.29E-10 |
| rs56258462 | G | A | -0.0209743 | 0.969366 | 0.0042169 | 6.58E-07 |
| rs6416745 | G | A | -0.0124135 | 0.0806641 | 0.0027017 | 4.34E-06 |
| rs72809020 | T | C | 0.0090508 | 0.816341 | 0.0018995 | 1.89E-06 |
| rs73112126 | C | T | 0.0301717 | 0.987254 | 0.0065788 | 4.52E-06 |
| rs76459067 | G | C | -0.0105862 | 0.875241 | 0.0022094 | 1.66E-06 |
| rs76585640 | G | C | -0.0165642 | 0.953589 | 0.0034601 | 1.69E-06 |
| rs77271157 | G | A | -0.0114995 | 0.894674 | 0.0024432 | 2.52E-06 |
| rs7870366 | A | G | -0.0115467 | 0.897557 | 0.0023935 | 1.41E-06 |
| rs9688032 | T | A | -0.0074914 | 0.706779 | 0.0016297 | 4.30E-06 |
|  |  |  |  |  |  |  |
| **Pure fruit juice** | |  |  |  |  |  |
| rs10048870 | G | T | 0.0081751 | 0.786349 | 0.0017865 | 4.74E-06 |
| rs117433925 | T | A | -0.0264908 | 0.980565 | 0.0056684 | 2.97E-06 |
| rs184067601 | A | G | 0.0208237 | 0.970781 | 0.0045349 | 4.40E-06 |
| rs191071605 | T | C | 0.0224009 | 0.976926 | 0.0049021 | 4.89E-06 |
| rs2043294 | A | T | -0.0088185 | 0.817401 | 0.0019089 | 3.85E-06 |
| rs4289676 | G | A | 0.0215837 | 0.975351 | 0.0047173 | 4.76E-06 |
| rs72563842 | T | C | -0.0110517 | 0.897937 | 0.0024091 | 4.49E-06 |
| rs9972653 | G | T | 0.0075914 | 0.598821 | 0.0014961 | 3.90E-07 |
| rs997800 | G | A | -0.0077504 | 0.711656 | 0.0016377 | 2.22E-06 |
|  |  |  |  |  |  |  |
| **Coffee** |  |  |  |  |  |  |
| rs10127720 | T | C | -0.0114265 | 0.25991 | 0.0017805 | 1.39E-10 |
| rs10193706 | A | C | -0.0076337 | 0.475168 | 0.0015677 | 1.12E-06 |
| rs10471885 | A | G | 0.0097749 | 0.796024 | 0.0019331 | 4.27E-07 |
| rs1057868 | C | T | -0.0186465 | 0.714671 | 0.0017213 | 2.44E-27 |
| rs10802317 | T | C | -0.0078731 | 0.636959 | 0.0016247 | 1.26E-06 |
| rs10853329 | T | C | -0.0081358 | 0.299114 | 0.0017457 | 3.16E-06 |
| rs10865548 | A | G | -0.0151198 | 0.171631 | 0.0020634 | 2.35E-13 |
| rs10938398 | G | A | -0.0072275 | 0.566682 | 0.0015764 | 4.55E-06 |
| rs10969937 | T | C | 0.0078145 | 0.679205 | 0.0016778 | 3.20E-06 |
| rs10992783 | C | T | 0.0075343 | 0.581738 | 0.0015825 | 1.93E-06 |
| rs10997940 | C | T | 0.008799 | 0.596495 | 0.0015894 | 3.09E-08 |
| rs111883241 | C | T | 0.0198366 | 0.965753 | 0.0042761 | 3.50E-06 |
| rs111994577 | C | G | -0.0148987 | 0.926199 | 0.0029979 | 6.71E-07 |
| rs11206378 | G | A | -0.0078692 | 0.356908 | 0.0016255 | 1.29E-06 |
| rs112133680 | C | T | -0.0131856 | 0.906335 | 0.0026732 | 8.12E-07 |
| rs114908993 | C | T | 0.0211006 | 0.967485 | 0.0044637 | 2.28E-06 |
| rs115109216 | C | G | 0.0176216 | 0.944812 | 0.003433 | 2.85E-07 |
| rs1153598 | A | G | -0.0113633 | 0.864999 | 0.0022759 | 5.95E-07 |
| rs116094457 | C | T | -0.0240125 | 0.971586 | 0.0046873 | 3.01E-07 |
| rs117810762 | G | A | -0.0348164 | 0.982026 | 0.0059424 | 4.66E-09 |
| rs117968677 | G | A | 0.0291003 | 0.975446 | 0.0052849 | 3.67E-08 |
| rs12201974 | C | T | -0.0094376 | 0.796094 | 0.00197 | 1.66E-06 |
| rs1260326 | T | C | -0.0130189 | 0.392804 | 0.0015921 | 2.92E-16 |
| rs12989746 | G | T | -0.0084317 | 0.749418 | 0.0017965 | 2.69E-06 |
| rs13054099 | T | C | 0.0091001 | 0.738767 | 0.0017705 | 2.75E-07 |
| rs13123752 | G | A | -0.0080364 | 0.385478 | 0.0015981 | 4.94E-07 |
| rs13378244 | G | A | -0.0081453 | 0.582265 | 0.0015809 | 2.57E-07 |
| rs144885044 | C | T | 0.0301421 | 0.985005 | 0.0065121 | 3.68E-06 |
| rs16903275 | C | A | -0.0150108 | 0.843641 | 0.0021427 | 2.46E-12 |
| rs17144566 | C | T | 0.0096984 | 0.805748 | 0.0019662 | 8.12E-07 |
| rs17687539 | A | G | 0.0104779 | 0.791308 | 0.001918 | 4.69E-08 |
| rs181624422 | C | T | 0.0220061 | 0.969537 | 0.0046598 | 2.33E-06 |
| rs1906251 | C | A | -0.0078765 | 0.356909 | 0.0016312 | 1.37E-06 |
| rs1956218 | A | G | -0.0076221 | 0.441168 | 0.0015732 | 1.27E-06 |
| rs2049019 | C | A | -0.0081515 | 0.313294 | 0.001683 | 1.28E-06 |
| rs2088568 | C | T | -0.0074432 | 0.407947 | 0.0015843 | 2.63E-06 |
| rs2231142 | G | T | 0.0148292 | 0.886419 | 0.0024495 | 1.42E-09 |
| rs2297508 | C | G | -0.0097861 | 0.349299 | 0.0016381 | 2.32E-09 |
| rs2330783 | G | T | 0.0434564 | 0.986115 | 0.0066456 | 6.20E-11 |
| rs2422370 | A | G | 0.0114297 | 0.821414 | 0.0022221 | 2.69E-07 |
| rs2465037 | C | A | 0.0107912 | 0.656857 | 0.0016462 | 5.56E-11 |
| rs2472297 | C | T | -0.0443926 | 0.733438 | 0.0017564 | 8.28E-141 |
| rs2494995 | A | C | 0.0091463 | 0.783055 | 0.0018895 | 1.29E-06 |
| rs2521501 | A | T | 0.009511 | 0.677292 | 0.0016777 | 1.44E-08 |
| rs2613458 | A | C | -0.0102541 | 0.278648 | 0.0017495 | 4.60E-09 |
| rs2979240 | C | G | -0.0079534 | 0.606484 | 0.0016079 | 7.56E-07 |
| rs3016390 | G | A | 0.0083528 | 0.619038 | 0.0016166 | 2.38E-07 |
| rs329124 | A | G | 0.0073568 | 0.580761 | 0.00158 | 3.22E-06 |
| rs34060476 | A | G | -0.017749 | 0.865801 | 0.0022851 | 8.05E-15 |
| rs34908430 | C | T | 0.0078821 | 0.700446 | 0.0016979 | 3.45E-06 |
| rs35189787 | C | T | -0.0091198 | 0.810332 | 0.0019853 | 4.36E-06 |
| rs395815 | C | A | 0.0096077 | 0.766411 | 0.0018397 | 1.77E-07 |
| rs4367098 | C | T | -0.0073988 | 0.378452 | 0.0016055 | 4.06E-06 |
| rs4410790 | T | C | -0.0378224 | 0.365536 | 0.001616 | 4.73E-121 |
| rs4475789 | T | G | -0.0084669 | 0.344545 | 0.0016408 | 2.47E-07 |
| rs4624274 | G | A | 0.0073815 | 0.568082 | 0.0015749 | 2.78E-06 |
| rs4697428 | C | T | -0.0129238 | 0.0930619 | 0.0026988 | 1.68E-06 |
| rs4877113 | A | G | -0.0075658 | 0.607683 | 0.0015948 | 2.10E-06 |
| rs55754437 | C | T | 0.0081933 | 0.72905 | 0.0017662 | 3.50E-06 |
| rs55780162 | A | G | -0.0074576 | 0.619668 | 0.001601 | 3.19E-06 |
| rs56113850 | T | C | -0.0120049 | 0.422625 | 0.0015747 | 2.47E-14 |
| rs57918684 | G | A | -0.012509 | 0.845143 | 0.0021561 | 6.57E-09 |
| rs586346 | T | C | 0.0095752 | 0.368843 | 0.0016179 | 3.26E-09 |
| rs597045 | A | T | 0.0101616 | 0.694903 | 0.0017109 | 2.86E-09 |
| rs6062682 | C | T | -0.0101876 | 0.534929 | 0.0015807 | 1.16E-10 |
| rs6124969 | C | T | -0.0086695 | 0.636038 | 0.0016207 | 8.83E-08 |
| rs62395028 | T | C | -0.0154287 | 0.941299 | 0.0033522 | 4.18E-06 |
| rs66723169 | C | A | -0.0140497 | 0.768639 | 0.0018522 | 3.32E-14 |
| rs72722581 | T | C | -0.0145072 | 0.905512 | 0.0026695 | 5.50E-08 |
| rs73075167 | A | T | 0.0157277 | 0.870337 | 0.0023547 | 2.41E-11 |
| rs75347775 | G | A | -0.0102908 | 0.754975 | 0.0018102 | 1.31E-08 |
| rs7580688 | A | G | -0.0087805 | 0.730047 | 0.0017513 | 5.34E-07 |
| rs763030 | G | C | 0.010945 | 0.855691 | 0.0022287 | 9.07E-07 |
| rs76450343 | C | T | -0.0271011 | 0.981763 | 0.005833 | 3.38E-06 |
| rs7664424 | C | G | 0.0074354 | 0.380108 | 0.0016146 | 4.12E-06 |
| rs7774229 | G | A | 0.01504 | 0.925296 | 0.002976 | 4.33E-07 |
| rs77936935 | G | A | 0.0231811 | 0.97137 | 0.0046729 | 7.03E-07 |
| rs7947502 | C | T | 0.0075599 | 0.40505 | 0.0015875 | 1.92E-06 |
| rs9729851 | C | T | -0.0082755 | 0.260034 | 0.0017719 | 3.01E-06 |
| rs9937053 | G | A | -0.0130711 | 0.577273 | 0.0015784 | 1.22E-16 |
| rs993885 | G | A | 0.0099019 | 0.633204 | 0.0016209 | 1.01E-09 |
|  |  |  |  |  |  |  |
| **Tea** |  |  |  |  |  |  |
| rs10305664 | A | C | -0.0100198 | 0.649102 | 0.0017914 | 2.23E-08 |
| rs10741694 | T | C | -0.0103637 | 0.373037 | 0.0017631 | 4.15E-09 |
| rs10906187 | A | G | -0.0095176 | 0.622882 | 0.0017596 | 6.35E-08 |
| rs10918630 | T | C | 0.0084612 | 0.315471 | 0.001838 | 4.15E-06 |
| rs10964538 | C | T | 0.010655 | 0.831166 | 0.0022742 | 2.80E-06 |
| rs11141683 | A | G | -0.0087566 | 0.6648 | 0.0018093 | 1.30E-06 |
| rs114278367 | A | G | 0.0279464 | 0.974894 | 0.0055152 | 4.04E-07 |
| rs115204443 | G | A | -0.030586 | 0.981555 | 0.0065844 | 3.40E-06 |
| rs1156588 | A | G | 0.0114927 | 0.790058 | 0.002093 | 4.00E-08 |
| rs11715828 | T | C | 0.0078557 | 0.519043 | 0.0017078 | 4.23E-06 |
| rs11752836 | A | G | -0.0079395 | 0.460902 | 0.0017132 | 3.58E-06 |
| rs12127737 | G | A | 0.0094739 | 0.221606 | 0.0020497 | 3.80E-06 |
| rs12190405 | T | A | 0.0098563 | 0.781927 | 0.0020905 | 2.42E-06 |
| rs12256016 | A | G | -0.0102793 | 0.736593 | 0.0019529 | 1.41E-07 |
| rs12347368 | C | T | 0.016161 | 0.927973 | 0.0032937 | 9.27E-07 |
| rs12410656 | C | T | 0.0174732 | 0.928158 | 0.003298 | 1.17E-07 |
| rs12446615 | A | G | 0.0086723 | 0.430616 | 0.001712 | 4.07E-07 |
| rs12518404 | T | C | -0.0081306 | 0.569028 | 0.001718 | 2.22E-06 |
| rs12554801 | C | T | -0.0090157 | 0.661934 | 0.0018073 | 6.09E-07 |
| rs12591786 | C | T | 0.0129231 | 0.842443 | 0.00237 | 4.96E-08 |
| rs13276110 | G | A | 0.0102441 | 0.772605 | 0.0020418 | 5.25E-07 |
| rs132904 | G | C | -0.0112523 | 0.220649 | 0.0020547 | 4.35E-08 |
| rs1368357 | C | T | -0.008314 | 0.653832 | 0.0017933 | 3.55E-06 |
| rs1421488 | G | A | -0.0085083 | 0.618878 | 0.0017574 | 1.29E-06 |
| rs1453548 | T | A | 0.0102908 | 0.335477 | 0.0018084 | 1.27E-08 |
| rs1481012 | A | G | 0.0154206 | 0.887422 | 0.002697 | 1.08E-08 |
| rs1529659 | C | T | 0.010373 | 0.179725 | 0.0022267 | 3.19E-06 |
| rs16930628 | G | A | -0.0117257 | 0.84948 | 0.0023884 | 9.13E-07 |
| rs17245213 | G | A | 0.0104689 | 0.792882 | 0.0021018 | 6.33E-07 |
| rs17280180 | C | G | -0.0084691 | 0.587529 | 0.0017309 | 9.94E-07 |
| rs17315220 | C | T | -0.0113416 | 0.849063 | 0.0023792 | 1.87E-06 |
| rs17539694 | G | C | -0.0087049 | 0.634498 | 0.0017671 | 8.39E-07 |
| rs17762577 | C | T | -0.008126 | 0.545707 | 0.0017071 | 1.93E-06 |
| rs1890389 | T | G | 0.0086493 | 0.464165 | 0.0017169 | 4.71E-07 |
| rs1973243 | T | C | -0.0105438 | 0.812797 | 0.0021883 | 1.45E-06 |
| rs2074551 | G | C | -0.0114957 | 0.725071 | 0.0020194 | 1.25E-08 |
| rs2117137 | A | G | -0.0116293 | 0.594668 | 0.0017326 | 1.92E-11 |
| rs2194507 | A | T | -0.0087238 | 0.357692 | 0.0018276 | 1.81E-06 |
| rs2234233 | G | A | -0.0114599 | 0.832458 | 0.0022764 | 4.80E-07 |
| rs2271961 | T | C | -0.0102064 | 0.500455 | 0.0017024 | 2.03E-09 |
| rs2273446 | C | G | -0.0141601 | 0.826923 | 0.0022668 | 4.20E-10 |
| rs2279844 | G | A | 0.0085608 | 0.623447 | 0.0017576 | 1.11E-06 |
| rs2350271 | T | A | -0.0174138 | 0.941002 | 0.0036663 | 2.04E-06 |
| rs2403304 | T | C | 0.008569 | 0.629087 | 0.0017626 | 1.16E-06 |
| rs2422370 | A | G | -0.0117134 | 0.821423 | 0.0024303 | 1.44E-06 |
| rs2472297 | C | T | -0.0311404 | 0.733412 | 0.0019221 | 5.20E-59 |
| rs2504716 | C | T | -0.0169458 | 0.791094 | 0.0020979 | 6.63E-16 |
| rs2659616 | T | C | -0.0079602 | 0.501788 | 0.0017071 | 3.12E-06 |
| rs2664404 | A | G | 0.0092188 | 0.432251 | 0.001724 | 8.94E-08 |
| rs2813703 | G | A | -0.0104569 | 0.428357 | 0.0017206 | 1.22E-09 |
| rs28691221 | C | T | -0.0085491 | 0.59974 | 0.0017439 | 9.47E-07 |
| rs290697 | G | T | -0.0082644 | 0.364633 | 0.0017686 | 2.97E-06 |
| rs2937676 | G | A | -0.0087012 | 0.296195 | 0.0018726 | 3.37E-06 |
| rs2940806 | T | C | 0.00789 | 0.458504 | 0.0017184 | 4.40E-06 |
| rs349356 | G | T | -0.009685 | 0.296672 | 0.0018701 | 2.23E-07 |
| rs34940743 | A | G | -0.0093244 | 0.652789 | 0.001784 | 1.73E-07 |
| rs35596618 | A | G | 0.0146291 | 0.91101 | 0.0029895 | 9.91E-07 |
| rs3939638 | T | C | 0.0118043 | 0.166448 | 0.0022962 | 2.74E-07 |
| rs4410790 | T | C | -0.0225102 | 0.365478 | 0.0017686 | 4.22E-37 |
| rs4468472 | T | C | -0.0082499 | 0.595763 | 0.0017433 | 2.22E-06 |
| rs4817505 | T | C | -0.012957 | 0.60831 | 0.0017478 | 1.23E-13 |
| rs4862411 | T | C | -0.0093038 | 0.235505 | 0.0020074 | 3.57E-06 |
| rs56163935 | T | C | -0.0145681 | 0.818619 | 0.0022128 | 4.59E-11 |
| rs56188862 | T | C | 0.0109031 | 0.614518 | 0.001752 | 4.87E-10 |
| rs57631352 | A | G | 0.0087528 | 0.70282 | 0.0018647 | 2.68E-06 |
| rs603826 | A | C | -0.0089386 | 0.629386 | 0.0017641 | 4.04E-07 |
| rs6070328 | T | C | 0.0084258 | 0.688963 | 0.001839 | 4.62E-06 |
| rs62296072 | T | C | -0.0162823 | 0.931943 | 0.003413 | 1.84E-06 |
| rs6462899 | T | A | -0.0096718 | 0.375431 | 0.0017629 | 4.11E-08 |
| rs6593661 | C | A | 0.0116134 | 0.134789 | 0.002495 | 3.25E-06 |
| rs713598 | C | G | -0.0105815 | 0.599989 | 0.0017366 | 1.11E-09 |
| rs7161654 | C | T | -0.0096021 | 0.292299 | 0.0018695 | 2.81E-07 |
| rs72797284 | A | G | 0.0119457 | 0.727523 | 0.0019119 | 4.16E-10 |
| rs72802733 | C | T | 0.0113531 | 0.862575 | 0.0024764 | 4.55E-06 |
| rs73487020 | T | G | -0.0136906 | 0.892034 | 0.002743 | 6.01E-07 |
| rs74672716 | G | A | -0.0104198 | 0.770126 | 0.0020258 | 2.70E-07 |
| rs76207439 | G | A | -0.0143304 | 0.91362 | 0.0030254 | 2.17E-06 |
| rs77123275 | C | T | -0.0179386 | 0.947794 | 0.0038661 | 3.48E-06 |
| rs7778305 | T | C | 0.0087815 | 0.478751 | 0.0017077 | 2.72E-07 |
| rs7799744 | C | T | -0.0142148 | 0.91466 | 0.003054 | 3.25E-06 |
| rs79494947 | G | A | -0.0194537 | 0.95294 | 0.0042615 | 5.00E-06 |
| rs9480167 | T | C | -0.0104547 | 0.78522 | 0.0020749 | 4.69E-07 |
| rs9551284 | G | A | -0.0087669 | 0.674477 | 0.0018256 | 1.57E-06 |
| rs960241 | C | G | 0.0085568 | 0.597466 | 0.0017395 | 8.69E-07 |
| rs9624470 | G | A | -0.0152164 | 0.419938 | 0.0017319 | 1.56E-18 |
| rs9866956 | A | G | 0.0096101 | 0.24065 | 0.0020008 | 1.56E-06 |
|  |  |  |  |  |  |  |
| **Alcoholic drinks** | |  |  |  |  |  |
| rs10043587 | A | G | -0.0114004 | 0.4794 | 0.0021027 | 4.80E-08 |
| rs1004787 | G | A | -0.0186852 | 0.468762 | 0.0021064 | 9.50E-19 |
| rs1022528 | G | A | -0.0129013 | 0.660611 | 0.0022169 | 3.30E-09 |
| rs11078696 | G | T | -0.0195712 | 0.195756 | 0.0027568 | 1.60E-12 |
| rs11125160 | G | A | -0.0123263 | 0.326969 | 0.0022378 | 3.50E-08 |
| rs11223617 | G | A | 0.0182231 | 0.79372 | 0.0025969 | 1.40E-12 |
| rs113441031 | C | T | 0.018031 | 0.829801 | 0.0027998 | 1.20E-10 |
| rs117782187 | C | T | -0.0302026 | 0.954843 | 0.0052244 | 1.50E-08 |
| rs11787216 | C | T | 0.0144554 | 0.629481 | 0.002213 | 7.40E-11 |
| rs118188698 | T | C | -0.0300974 | 0.944909 | 0.0047003 | 2.20E-10 |
| rs11864886 | A | G | -0.0175254 | 0.774939 | 0.0025082 | 2.50E-12 |
| rs11940694 | A | G | -0.0357367 | 0.394614 | 0.0021608 | 6.50E-62 |
| rs12030672 | C | A | -0.013101 | 0.755869 | 0.0024336 | 4.90E-08 |
| rs12184585 | A | G | -0.0128822 | 0.287097 | 0.0023381 | 3.80E-08 |
| rs1229984 | T | C | -0.226432 | 0.0250629 | 0.0067513 | 3.80E-248 |
| rs12521723 | A | T | 0.0156981 | 0.836597 | 0.0028439 | 3.70E-08 |
| rs12593149 | G | A | -0.0123029 | 0.563285 | 0.0021231 | 1.20E-08 |
| rs1260326 | T | C | -0.0361951 | 0.39494 | 0.0021392 | 2.60E-64 |
| rs13024996 | C | A | 0.0141822 | 0.629828 | 0.0021655 | 6.20E-11 |
| rs13135092 | A | G | 0.0428616 | 0.916695 | 0.0038091 | 3.30E-29 |
| rs13390019 | T | C | 0.0194208 | 0.866796 | 0.003117 | 5.70E-10 |
| rs138774354 | G | C | 0.013002 | 0.61753 | 0.0022067 | 4.80E-09 |
| rs1397132 | T | C | 0.0115209 | 0.426749 | 0.0021236 | 3.70E-08 |
| rs149971103 | A | G | -0.0519513 | 0.983342 | 0.00827 | 3.30E-10 |
| rs17177078 | C | T | 0.0273857 | 0.94376 | 0.0045533 | 2.30E-09 |
| rs17601612 | G | C | 0.0147984 | 0.616346 | 0.0021731 | 8.00E-12 |
| rs1788030 | C | T | 0.0150956 | 0.541098 | 0.0021195 | 1.50E-12 |
| rs1788820 | A | G | -0.0158619 | 0.345675 | 0.0022003 | 7.50E-13 |
| rs1937522 | A | G | 0.0147987 | 0.471636 | 0.0020972 | 9.70E-13 |
| rs194868 | T | C | -0.0140053 | 0.540501 | 0.0020987 | 2.30E-11 |
| rs2079227 | G | A | -0.0119639 | 0.530439 | 0.0021024 | 9.10E-09 |
| rs2274793 | C | T | 0.0135923 | 0.672514 | 0.0022377 | 1.50E-09 |
| rs2525570 | A | G | -0.0119675 | 0.397872 | 0.0021405 | 1.60E-08 |
| rs2533196 | G | A | -0.0137467 | 0.533611 | 0.0021321 | 8.30E-11 |
| rs2717063 | C | A | -0.014175 | 0.414783 | 0.0021326 | 2.50E-11 |
| rs2858088 | A | G | -0.0147637 | 0.381833 | 0.0021577 | 1.00E-11 |
| rs28601761 | C | G | -0.0119457 | 0.580639 | 0.0021462 | 4.20E-08 |
| rs28929474 | C | T | 0.0473655 | 0.980049 | 0.0074983 | 1.70E-10 |
| rs2959005 | C | T | -0.0135182 | 0.330265 | 0.0022274 | 1.00E-09 |
| rs322776 | T | G | -0.0116123 | 0.420528 | 0.0021287 | 4.40E-08 |
| rs34060476 | A | G | -0.0207444 | 0.866331 | 0.0030797 | 3.20E-11 |
| rs35572189 | G | A | -0.0135756 | 0.637299 | 0.0021852 | 3.60E-10 |
| rs4284578 | T | C | 0.0123591 | 0.368093 | 0.0021731 | 1.60E-08 |
| rs4480324 | A | G | 0.0157159 | 0.294361 | 0.0023215 | 2.30E-11 |
| rs4726481 | G | T | 0.0131318 | 0.600177 | 0.002146 | 7.80E-10 |
| rs4733067 | A | G | -0.0118284 | 0.405014 | 0.0021322 | 3.40E-08 |
| rs4815364 | G | A | -0.0123534 | 0.393915 | 0.0021517 | 6.70E-09 |
| rs4844947 | G | C | 0.0125219 | 0.401258 | 0.0021371 | 2.40E-09 |
| rs4916723 | A | C | 0.0151795 | 0.578081 | 0.0021441 | 2.40E-12 |
| rs55932213 | A | G | -0.0141131 | 0.255131 | 0.0024491 | 1.10E-08 |
| rs55938136 | A | G | 0.023831 | 0.775012 | 0.0025071 | 2.10E-21 |
| rs56030824 | G | A | 0.018133 | 0.67822 | 0.0022412 | 3.10E-16 |
| rs56094641 | A | G | 0.0137086 | 0.595679 | 0.0021339 | 9.40E-11 |
| rs6136465 | G | A | 0.0123024 | 0.595652 | 0.002148 | 9.80E-09 |
| rs61873510 | G | T | 0.0157739 | 0.672217 | 0.0022886 | 3.40E-12 |
| rs62136829 | C | T | 0.0327803 | 0.949942 | 0.0048499 | 1.50E-11 |
| rs62244890 | T | C | -0.0127447 | 0.585228 | 0.0021265 | 1.70E-09 |
| rs62305780 | C | G | -0.0347147 | 0.897233 | 0.0034991 | 6.10E-23 |
| rs62442924 | C | T | -0.0177486 | 0.806658 | 0.002659 | 4.90E-11 |
| rs62477431 | C | G | 0.0146334 | 0.78302 | 0.0025657 | 1.50E-08 |
| rs6759325 | C | G | -0.0159196 | 0.842781 | 0.0028757 | 3.30E-08 |
| rs6810396 | C | G | -0.01507 | 0.804209 | 0.002643 | 1.70E-08 |
| rs6937318 | T | C | 0.0118074 | 0.492833 | 0.0020963 | 3.70E-08 |
| rs72890684 | C | A | 0.0287204 | 0.957366 | 0.0052227 | 3.20E-08 |
| rs74424378 | T | G | 0.0144413 | 0.76309 | 0.0024653 | 3.20E-09 |
| rs75746223 | C | T | 0.0412545 | 0.977435 | 0.0070704 | 3.80E-09 |
| rs7630012 | A | G | 0.0126553 | 0.565498 | 0.0021102 | 2.80E-09 |
| rs77123275 | C | T | -0.0286258 | 0.948114 | 0.0047689 | 1.30E-09 |
| rs7861804 | A | G | -0.0119897 | 0.545528 | 0.0022113 | 2.40E-08 |
| rs828867 | G | A | -0.0134357 | 0.457973 | 0.002137 | 2.80E-10 |
| rs838145 | G | A | 0.0151525 | 0.457726 | 0.0021206 | 9.80E-13 |
| rs911475 | C | T | 0.0157555 | 0.83277 | 0.0028354 | 2.80E-08 |
| rs928736 | C | T | 0.0141619 | 0.306626 | 0.0022938 | 4.20E-10 |
| rs9349379 | A | G | -0.0144514 | 0.594495 | 0.0021332 | 1.10E-11 |
| rs9597846 | A | G | 0.0164042 | 0.836381 | 0.002833 | 5.80E-09 |
| rs9607812 | G | A | 0.0154943 | 0.811563 | 0.0027002 | 7.40E-09 |
| rs9866322 | T | C | -0.0206574 | 0.773828 | 0.0025189 | 2.60E-16 |

SNP, single nucleotide polymorphism; EAF, Effect allele frequency; SE, Standard error.

# Table S11. Mendelian randomization estimates for the causal associations between sugar sweetened beverages consumption and estrogen receptor (ER)-positive/negative breast cancer

| **Outcome** | **SNP** | **F statistic** | **Methods** | **OR (95% CI)** | ***P*** | **Cochran’s  Q statistic (I^2^)** | **MR-Egger  Intercept (*P*)** |
| --- | --- | --- | --- | --- | --- | --- | --- |
|  |  |  |  |  |  |  |  |
| ER-positive breast cancer | 14 | 24.1 | IVW | 3.07 (0.94, 9.98) | 0.06 | 57.92 (77.6%) | 0.008 (0.67) |
|  |  |  | WM | 1.78 (0.78, 4.07) | 0.17 |  |  |
|  |  |  | MR-Egger | 1.45 (0.04, 52.70) | 0.84 |  |  |
|  |  |  | MR-PRESSO* | 1.43 (0.82, 2.48) | 0.23 |  |  |
| ER-negative breast cancer | 14 | 24.1 | IVW | 5.69 (1.22, 26.63) | 0.03 | 42.26 (69.2%) | 0.004 (0.88) |
|  |  |  | WM | 2.95 (0.86, 10.16) | 0.09 |  |  |
|  |  |  | MR-Egger | 4.05 (0.03, 470.05) | 0.57 |  |  |
|  |  |  | MR-PRESSO* | 2.14 (0.97, 4.75) | 0.08 |  |  |

Abbreviation: ER, estrogen receptor; SNP, single nucleotide polymorphism; IVW, inverse-variance weighted; WM, weighted median method; MR-PRESSO, Mendelian randomization pleiotropy residual sum and outlier; OR, odds ratio; CI, confidence interval.

*1 SNP was identified as influential outliers when ER-positive breast cancer was outcome: rs55872725; 1 SNP was identified as influential outliers when ER-negative breast cancer was outcome: rs55872725.

# Table S12. Mendelian randomization estimates for the causal associations between sugar sweetened beverages consumption and anthropo-metabolic biomarkers

| **Anthropo-metabolic biomarker** | **SNP** | **F statistic** | **β (95% CI) ^a^** | ***P*** | **Cochran’s  Q statistic (I^2^)** | **MR-Egger  Intercept (*P*)** |
| --- | --- | --- | --- | --- | --- | --- |
|  |  |  |  |  |  |  |
| Body mass index | 4 | 23.3 | -0.25 (-0.76, 0.26) | 0.33 | 1.06 (0.0%) | -0.004 (0.64) |
| Waist circumference | 4 | 23.3 | -0.06 (-0.64, 0.52) | 0.84 | 2.65 (0.0%) | -0.006 (0.62) |
| Waist-to-hip ratio | 4 | 23.3 | -0.26 (-0.96, 0.43) | 0.46 | 4.52 (33.7%) | -0.015 (0.21) |
| Fasting glucose | 14 | 24.1 | 0.04 (-0.09, 0.18) | 0.55 | 12.73 (0.0%) | 0.002 (0.42) |
| Acetone | 14 | 24.1 | -0.05 (-0.35, 0.25) | 0.74 | 13.55 (4.1%) | -0.001 (0.88) |
| Serum total cholesterol | 14 | 24.1 | 0.02 (-0.30, 0.35) | 0.90 | 16.83 (22.8%) | -0.003 (0.55) |
| LDL-C | 14 | 24.1 | -0.05 (-0.42, 0.31) | 0.79 | 21.13 (38.5%) | -0.004 (0.50) |
| HDL-C | 14 | 24.1 | 0.39 (0.10, 0.67) | 0.008 | 9.84 (0.0%) | 0.004 (0.33) |
| Serum total triglycerides | 14 | 24.1 | -0.34 (-0.68, -0.01) | 0.04 | 17.46 (25.6%) | -0.003 (0.58) |
| Total fatty acids | 14 | 24.1 | -0.23 (-0.55, 0.08) | 0.15 | 15.53 (16.3%) | -0.004 (0.40) |
| Monounsaturated fatty acids | 14 | 24.1 | -0.21 (-0.50, 0.08) | 0.15 | 11.14 (0.0%) | -0.005 (0.30) |
| Ratio of monounsaturated fatty acids to TFAs | 14 | 24.1 | -0.15 (-0.44, 0.13) | 0.30 | 8.26 (0.0%) | -0.003 (0.54) |
| Polyunsaturated fatty acids | 14 | 24.1 | -0.06 (-0.43, 0.31) | 0.75 | 21.67 (40.0%) | -0.002 (0.70) |
| Ratio of polyunsaturated fatty acids to TFAs | 14 | 24.1 | 0.37 (0.09, 0.66) | 0.01 | 12.32 (0.0%) | 0.002 (0.64) |
| Ratio of omega-3 fatty acids to TFAs | 14 | 24.1 | 0.19 (-0.09, 0.48) | 0.18 | 11.37 (0.0%) | -0.005 (0.28) |
| Ratio of omega-6 fatty acids to TFAs | 14 | 24.1 | 0.36 (0.07, 0.66) | 0.02 | 10.21 (0.0%) | 0.004 (0.42) |
| Bilirubin | 10 | 25.0 | 0.03 (-0.39, 0.46) | 0.87 | 9.12 (1.3%) | -0.008 (0.22) |
| Urea | 14 | 24.1 | -0.10 (-1.94, 1.74) | 0.92 | 36.61 (64.5%) | -0.004 (0.90) |
| Creatinine | 14 | 24.1 | 0.23 (-1.08, 1.54) | 0.73 | 23.29 (44.2%) | -0.004 (0.87) |
| Urate | 3 | 24.0 | 1.20 (-1.16, 3.55) | 0.32 | 14.27 (86.0%) | 0.046 (0.35) |
| Valine | 14 | 24.1 | 0.30 (-0.94, 1.54) | 0.63 | 16.04 (19.0%) | -0.024 (0.21) |
| Lysine | 14 | 24.1 | 0.35 (-1.02, 1.73) | 0.62 | 19.10 (31.9%) | 0.009 (0.69) |
| Arginine | 14 | 24.1 | 0.01 (-1.17, 1.20) | 0.98 | 14.20 (8.4%) | -0.004 (0.82) |
| Glutamine | 14 | 24.1 | -0.36 (-1.98, 1.27) | 0.67 | 26.52 (51.0%) | -0.020 (0.44) |
| Creatine | 14 | 24.1 | -0.27 (-1.51, 0.97) | 0.67 | 18.45 (29.5%) | 0.001 (0.96) |

Abbreviations: LDL-C, low-density lipoprotein cholesterol; HDL-C, high-density lipoprotein cholesterol; TFAs, total fatty acids; SNP, single nucleotide polymorphism; CI, confidence interval.

^a^ The β and 95% CI were estimated using two-sample univariable Mendelian randomization analysis, with inverse-variance weighted as the main method.

# Table S13. Mendelian randomization estimates for the causal associations between anthropo-metabolic biomarkers and overall breast cancer

| **Mediator** | **SNP** | **F statistic** | **OR (95% CI) ^a^** | ***P*** | **Cochran’s  Q statistic (I^2^)** | **MR-Egger  Intercept (*P*)** |
| --- | --- | --- | --- | --- | --- | --- |
|  |  |  |  |  |  |  |
| HDL-C | 80 | 121.4 | 1.08 (1.03, 1.14) | 0.001 | 178.96 (55.9%) | 0.001 (0.74) |
| Serum total triglycerides | 68 | 141.1 | 0.96 (0.90, 1.02) | 0.18 | 238.39 (71.9%) | 0.008 (0.005) |
| Ratio of polyunsaturated fatty acids to TFAs | 38 | 110.7 | 1.10 (1.02, 1.18) | 0.02 | 93.69 (60.5%) | 0.003 (0.53) |
| Ratio of omega-6 fatty acids to TFAs | 32 | 114.5 | 1.13 (1.05, 1.22) | 0.001 | 70.19 (55.8%) | 0.007 (0.06) |

Abbreviations: HDL-C, high-density lipoprotein cholesterol; TFAs, total fatty acids; SNP, single nucleotide polymorphism; CI, confidence interval.

^a^ The OR and 95% CI were estimated using two-sample univariable Mendelian randomization analysis, with inverse-variance weighted as the main method.


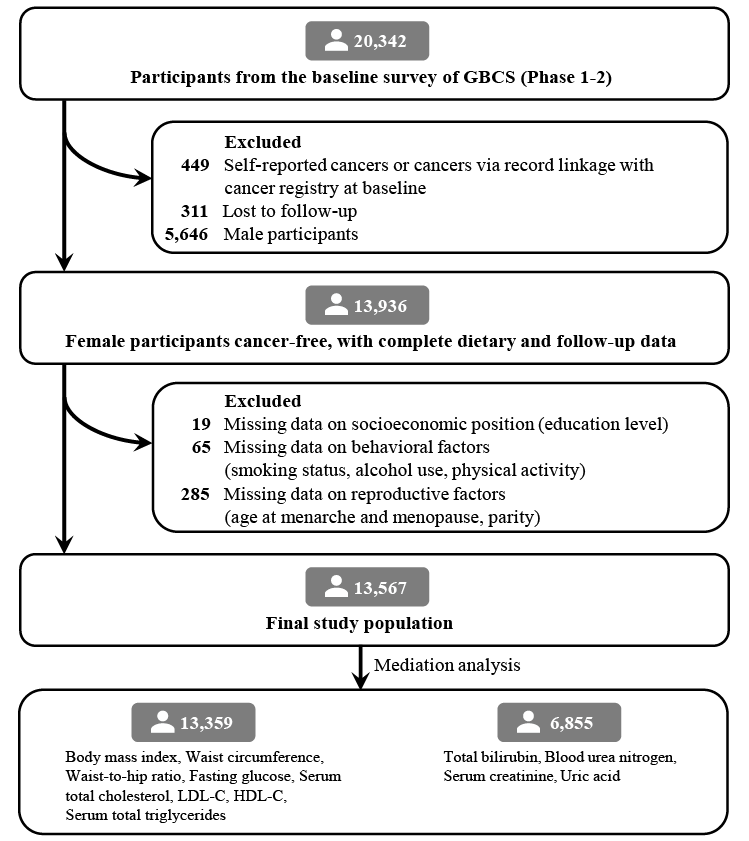


# Figure S1. Flow diagram of participants selection

Abbreviations: GBCS, Guangzhou Biobank Cohort Study; LDL, low-density lipoprotein; HDL-C, high-density lipoprotein cholesterol.
